# Supplementary material for: Highly hydrated paramagnetic amorphous calcium carbonate nanoclusters as an MRI contrast agent
Source: Nat Commun. 2022 Aug 29;13:5088. doi: 10.1038/s41467-022-32615-3 (PMC9424530; doi:10.1038/s41467-022-32615-3)
Supplement: Supplementary file 1 — Supplementary Information [file 41467_2022_32615_MOESM1_ESM.docx]

Supplementary Information

**Highly Hydrated Paramagnetic Amorphous Calcium Carbonate Nanoclusters as an MRI Contrast Agent**

Dong et al.


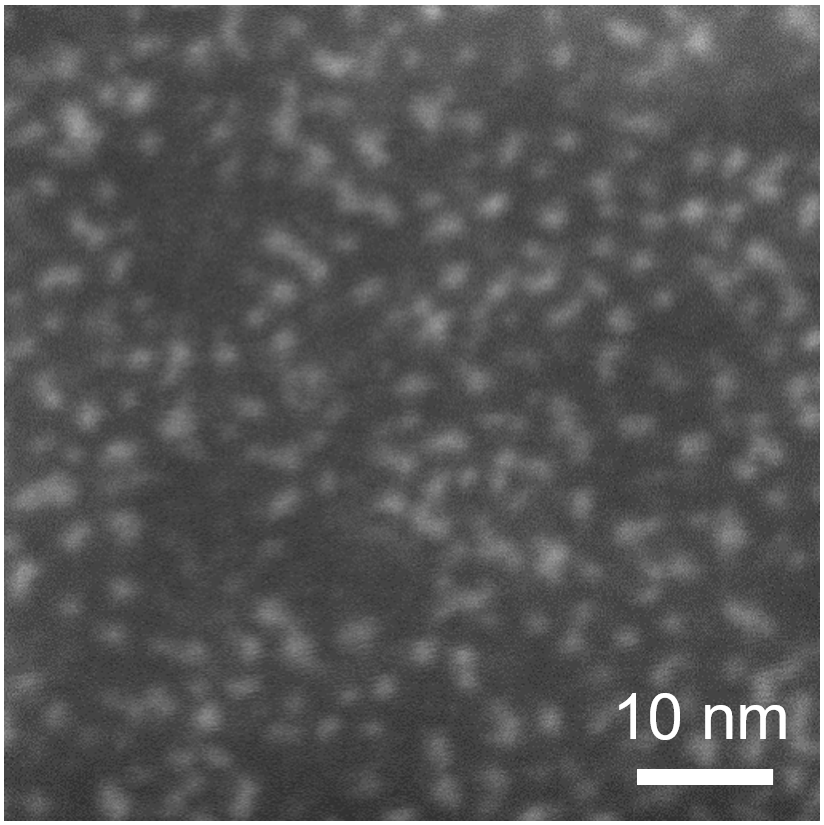


**Supplementary Figure 1. Characterization of the STEM image of ACNC.** STEM image of ACNC dispersed in normal saline solution. A representative image of three individual experiments is shown.


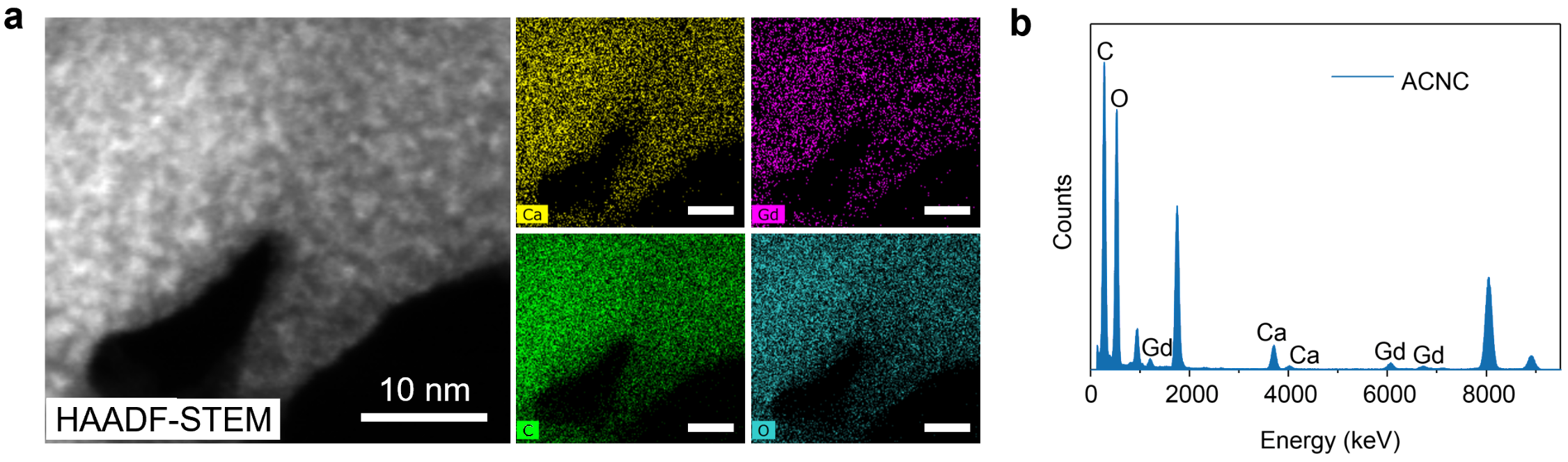


**Supplementary Figure 2. Characterization of the EDS results of ACNC. a** EDS mapping and **b** EDS spectrum of ACNC collected by ethanol precipitation. The experiments were repeated three times independently.


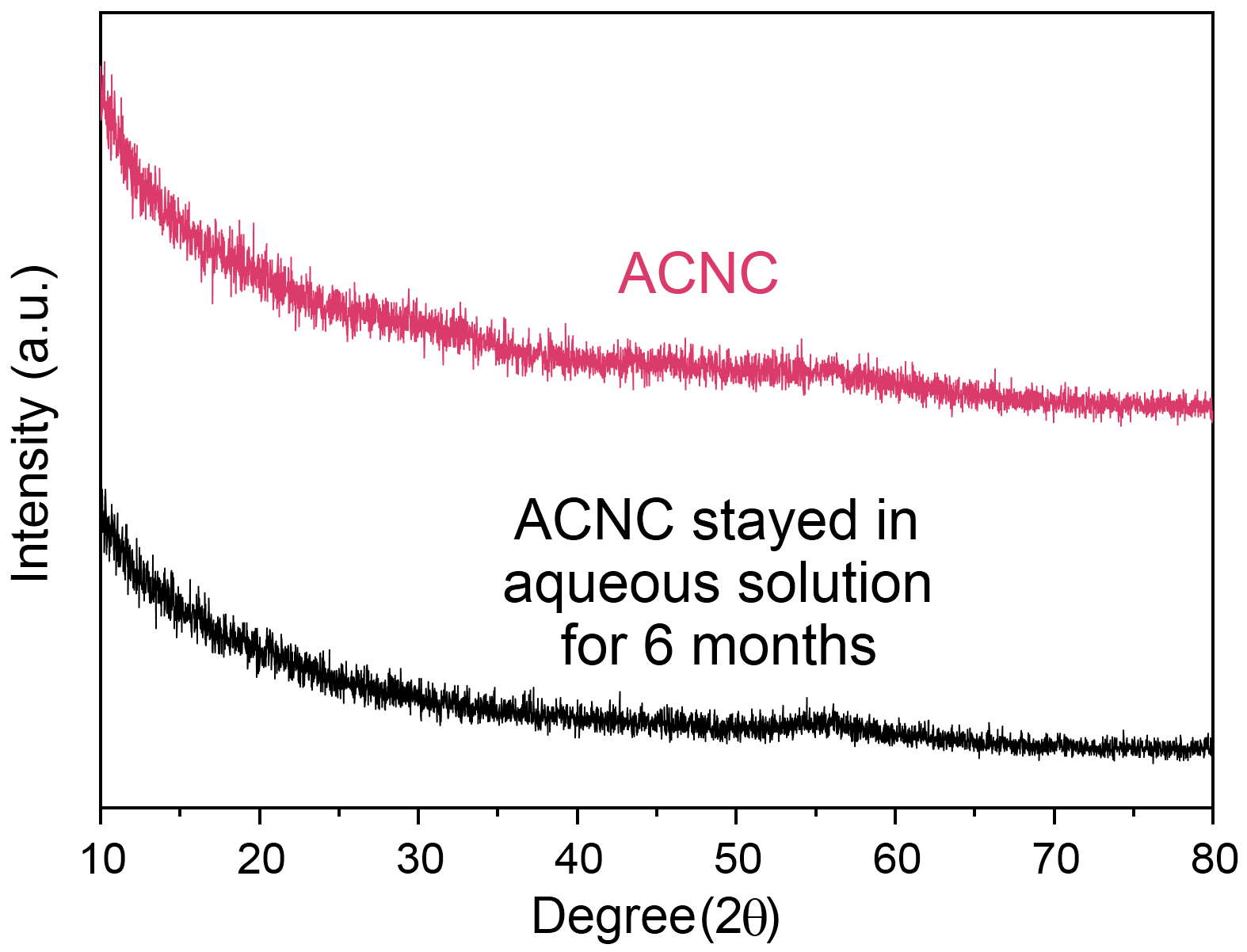


**Supplementary Figure 3. Characterization of the XRD results of ACNC.** XRD patterns of ACNC and ACNC stayed in aqueous solution for 6 months.


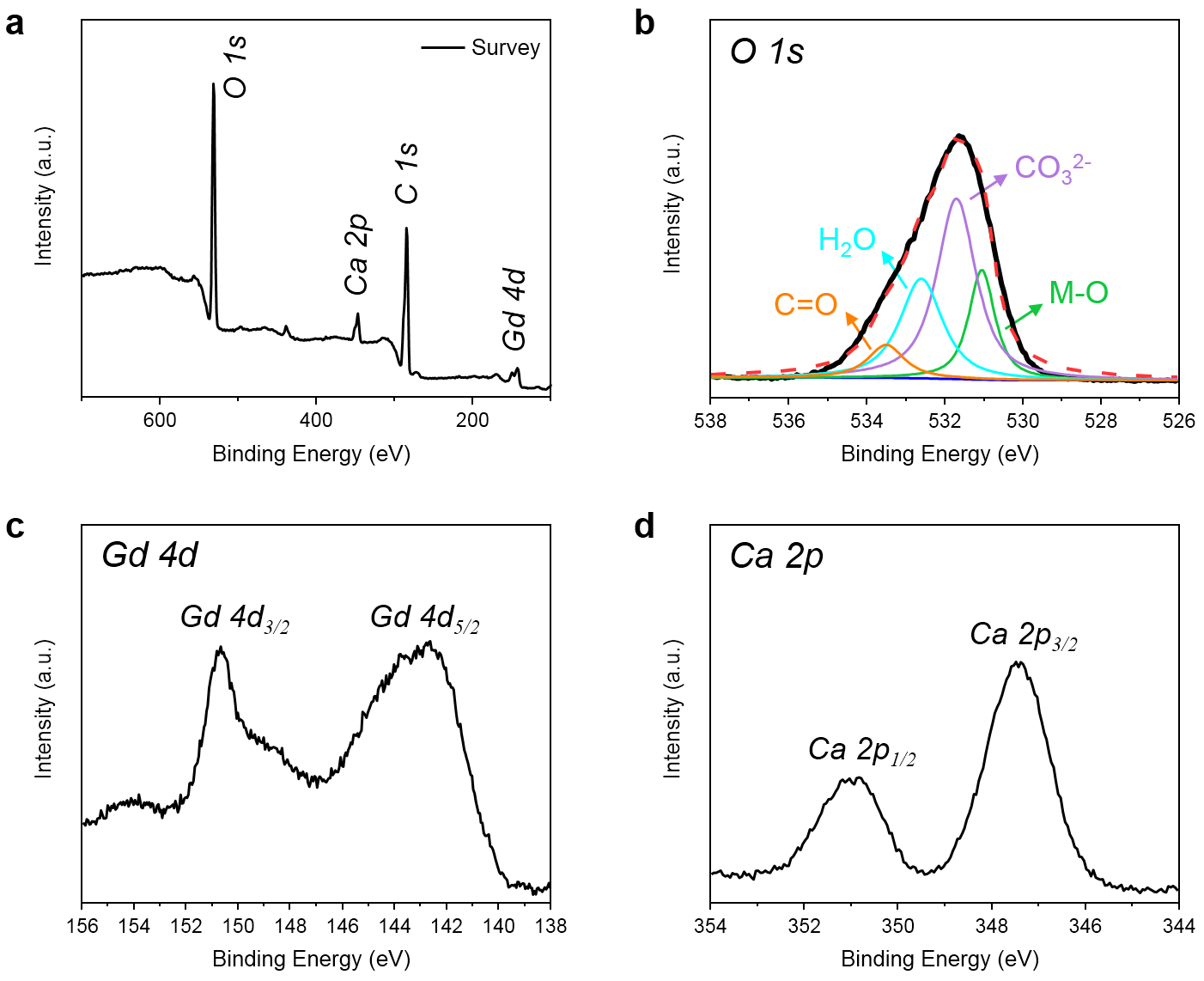


**Supplementary Figure 4. Characterization of the XPS results of ACNC. a** XPS survey spectrum of ACNC, and the corresponding spectrum of **b** *O 1s*, **c** *Gd 4d* and **d** *Ca 2p*. Black solid line and red dashed line corresponded to the raw and fitted curve, respectively.


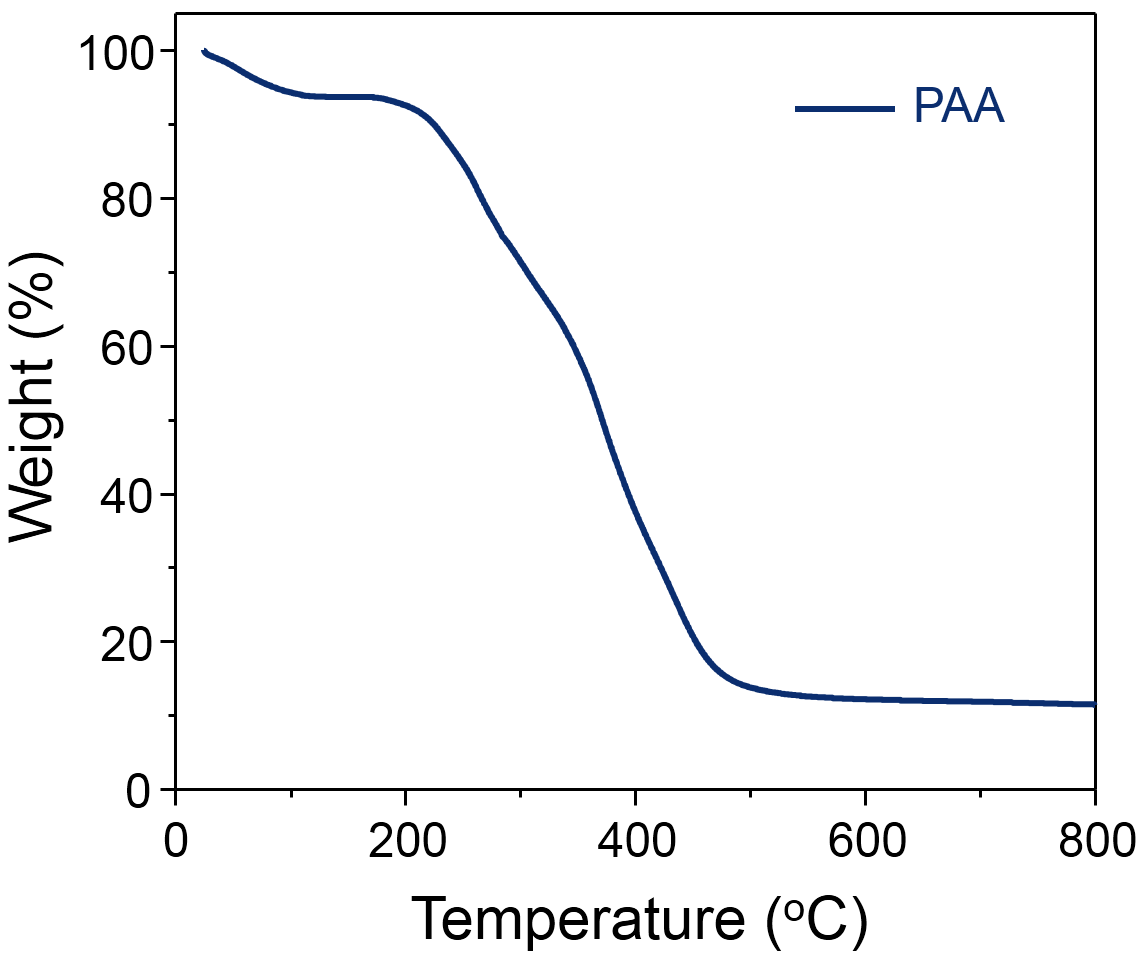


**Supplementary Figure 5. Characterization of the TG result of PAA.** Thermogravimetric curve of PAA powder under an N_2_ atmosphere with a heating rate of 10 °C min^-1^


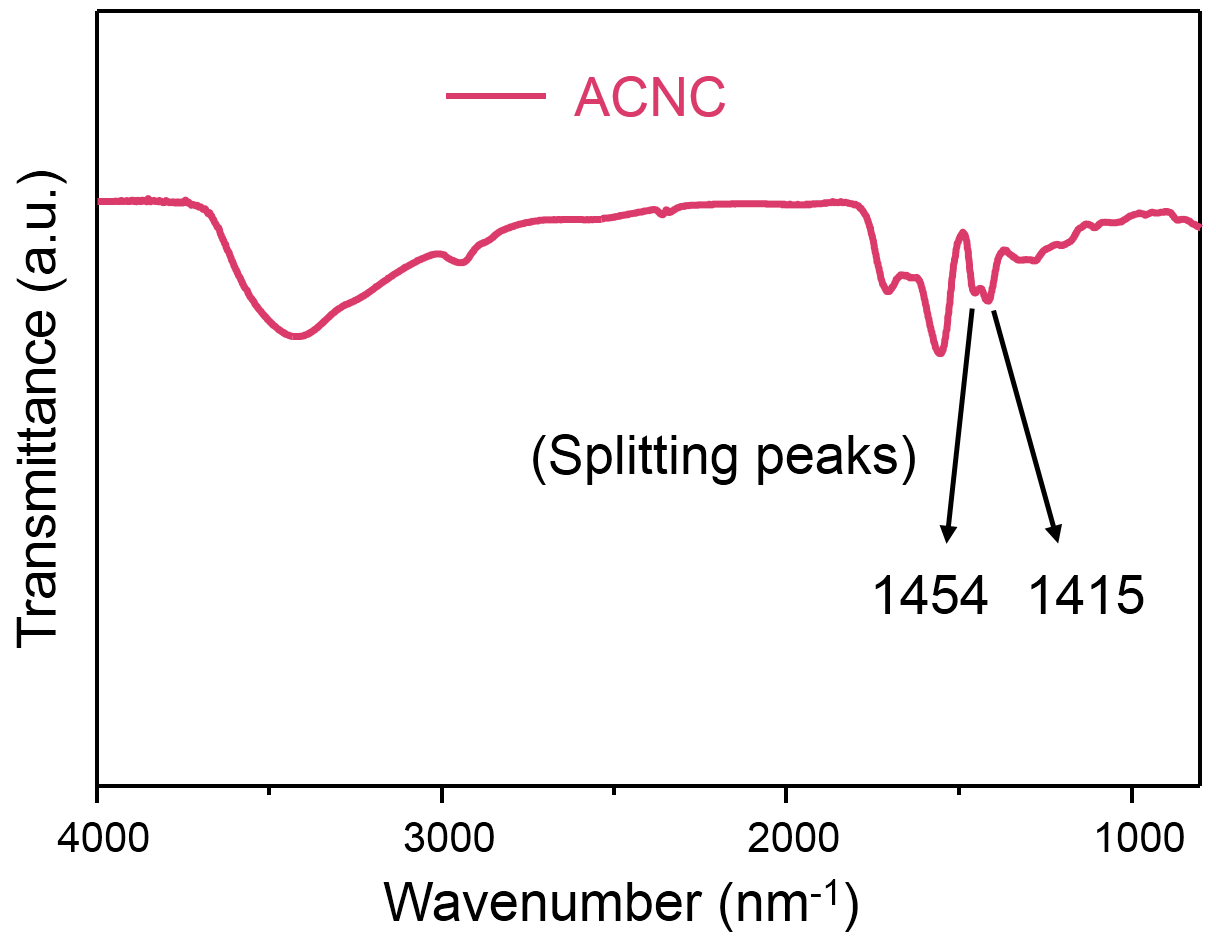


**Supplementary Figure 6. Characterization of the FT-IR result of ACNC.** FT-IR spectrum of ACNC. The split band at 1415 and 1454 cm^-1^ in the FT-IR spectrum can be assigned to the asymmetric stretch vibration of the carbonate ions in typical ACC environments.


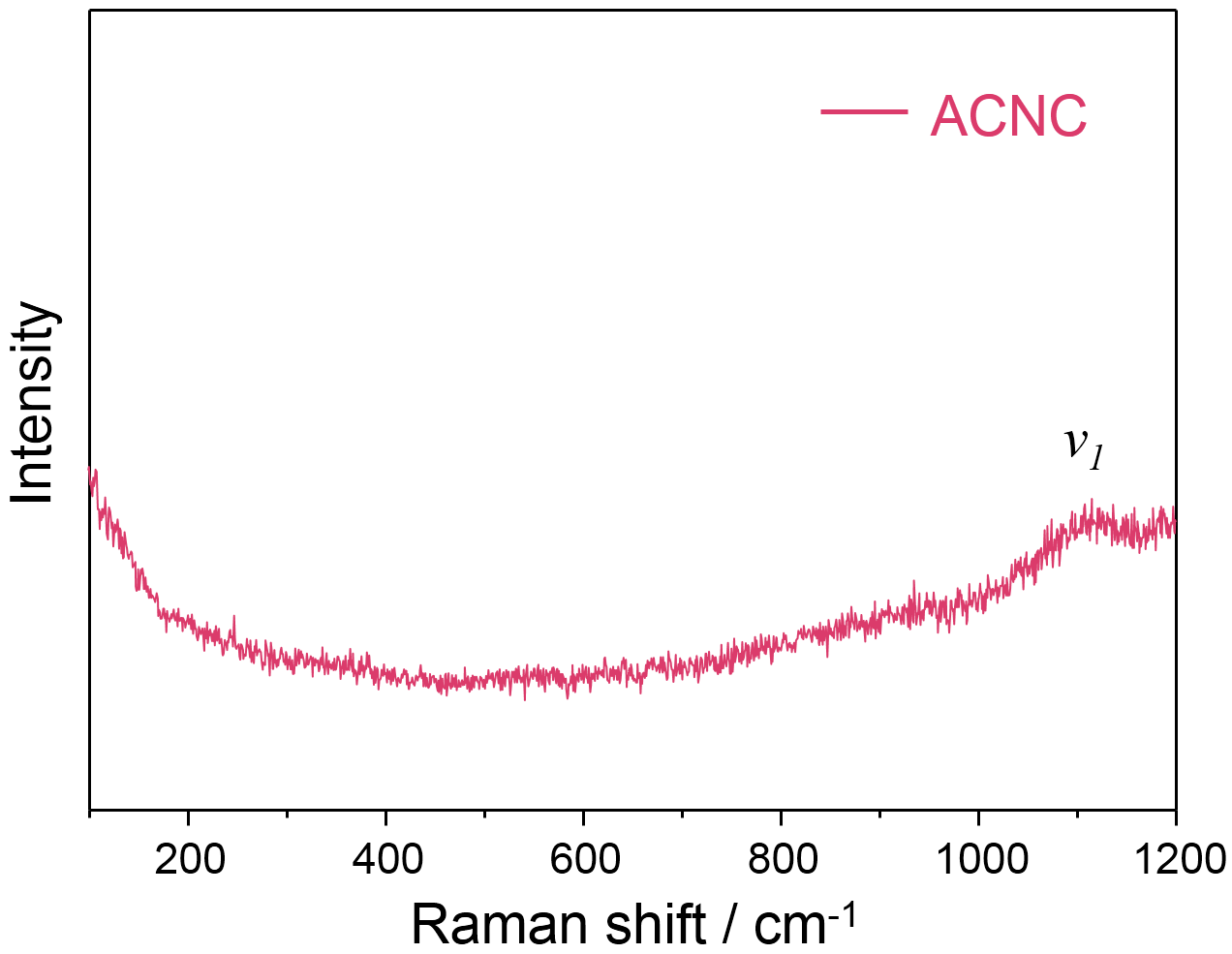


**Supplementary Figure 7. Characterization of the Raman result of ACNC.** Raman spectrum of ACNC. A broad peak centered at 1086 cm^-1^ was attributed to the internal CO_3_^2-^ symmetric stretch.


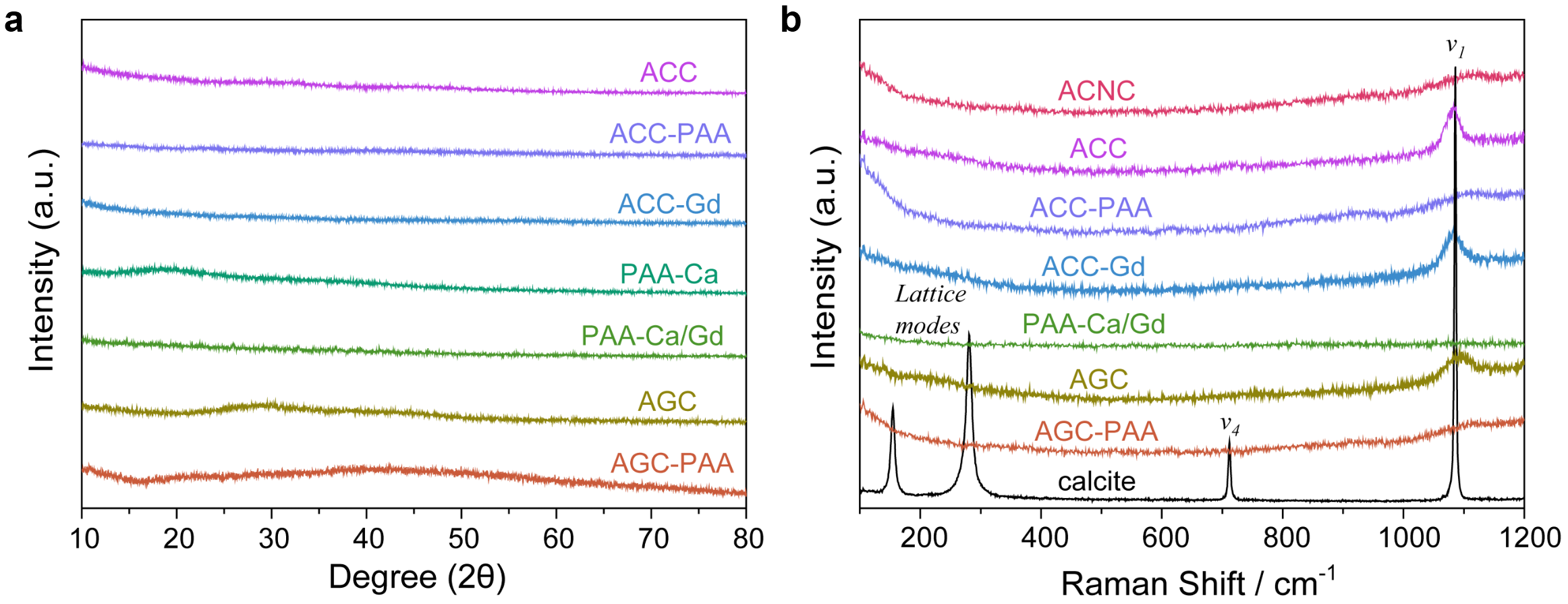


**Supplementary Figure 8. Characterization of the XRD and Raman results of a series of control samples.** **a** XRD patterns of ACNC and a series of control samples. **b** Raman spetra of ACNC and a series of control samples compared with calcite.


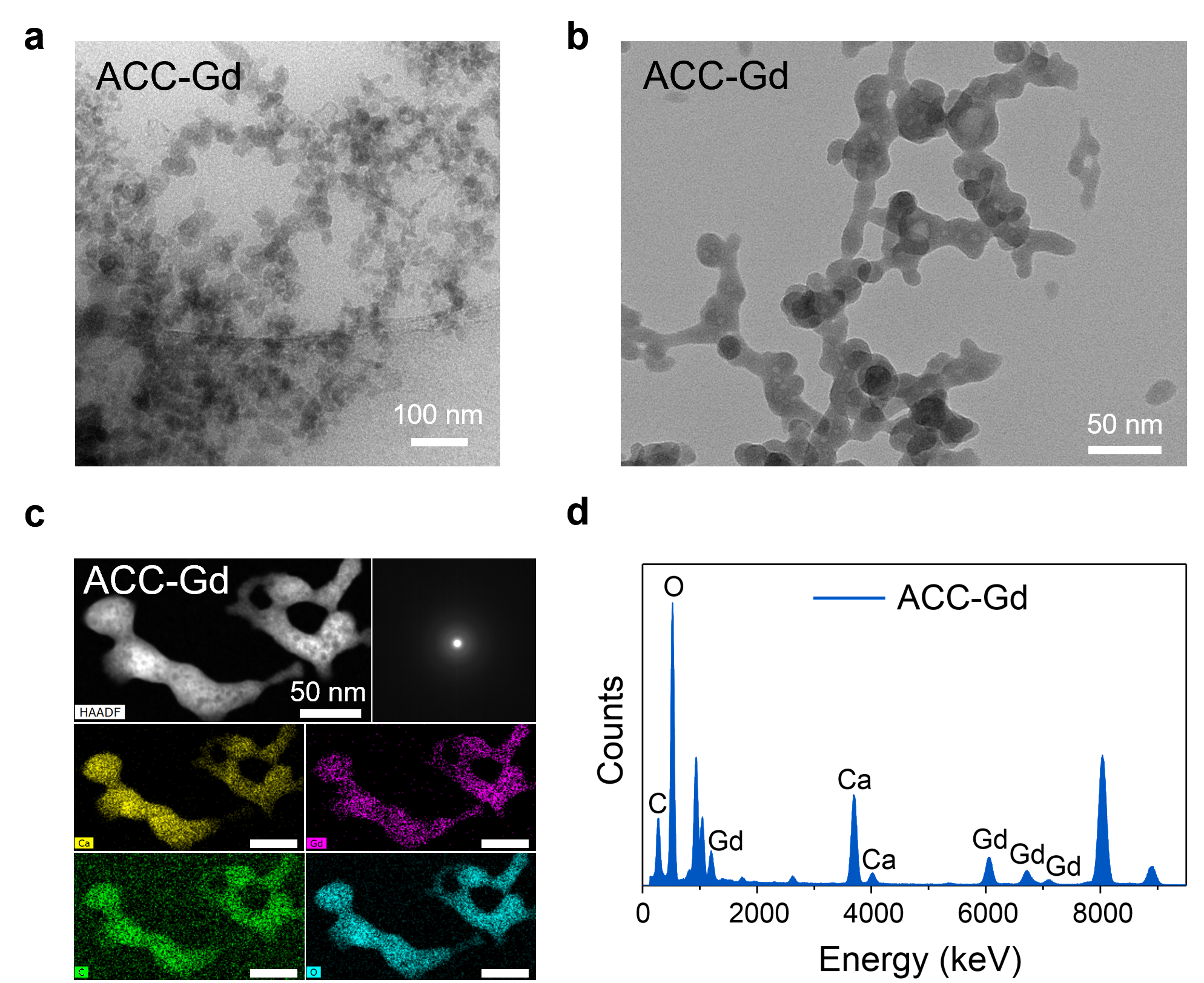


**Supplementary Figure 9. Characterization of the** **cryo-TEM image, TEM image and EDS results of ACC-Gd.** **a** cryo-TEM and **b** TEM image of ACC-Gd. A representative image of three individual experiments is shown. **c** HAADF-STEM image and SAED of ACC-Gd, and EDS mapping indicated the distribution of calcium (yellow), gadolinium (purple), carbon (green) and oxygen (cyan). A representative image of three individual experiments is shown. **d** EDS result of ACC-Gd.


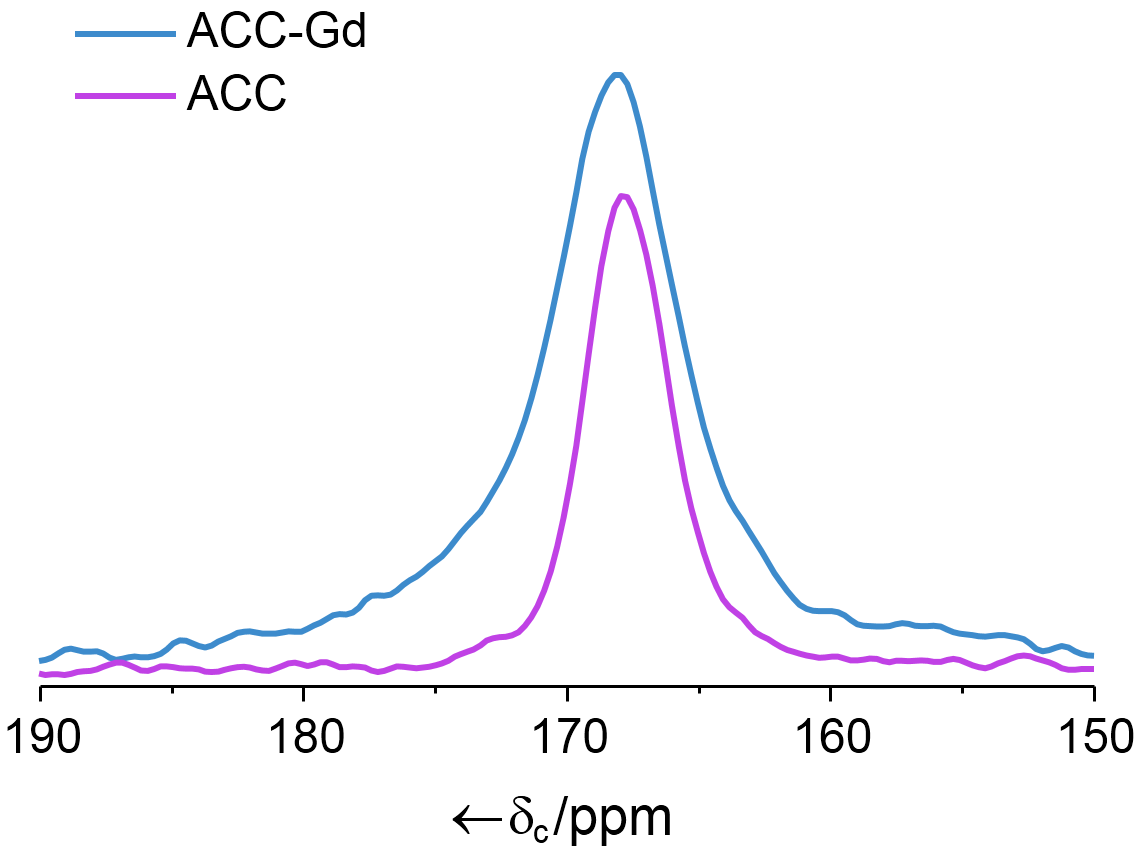


**Supplementary Figure 10. Characterization of the NMR spectra.** ^13^C solid-state NMR spectra of ACC-Gd and ACC recorded by hpdec spectra.


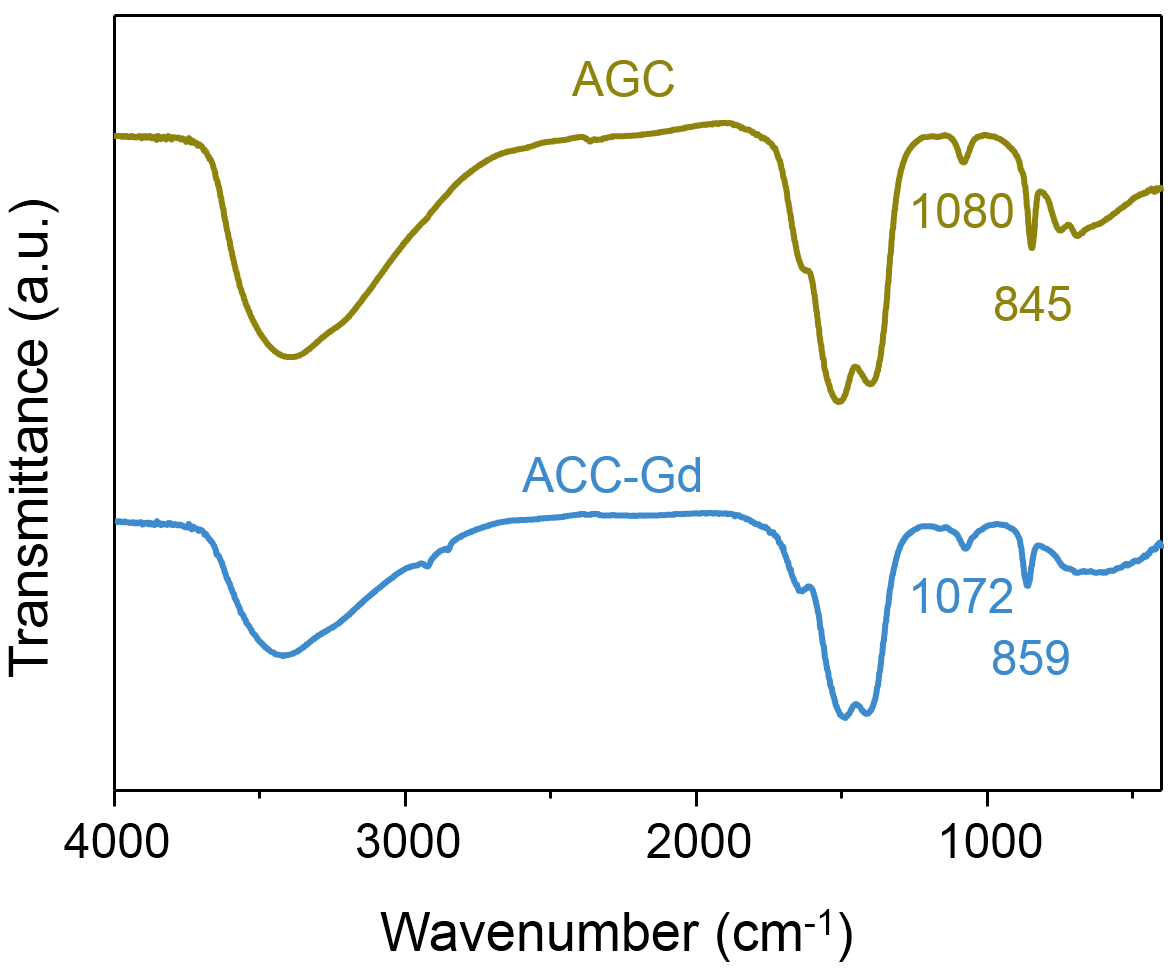


**Supplementary Figure 11. Characterization of the FT-IR spectra.** FT-IR spectra of amorphous gadolinium carbonate (AGC) and ACC-Gd.


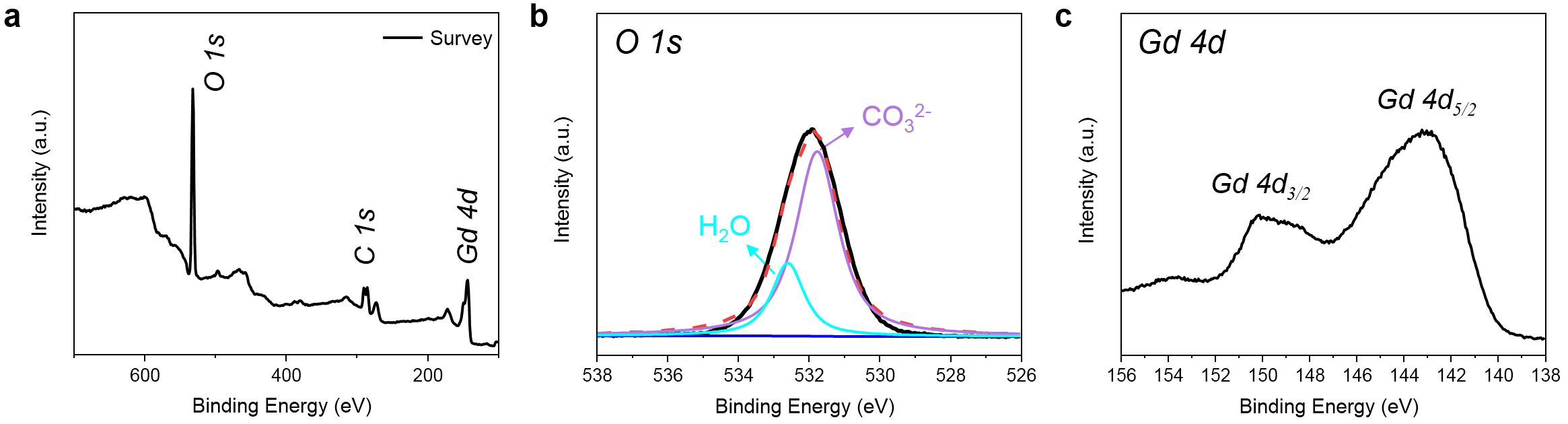


**Supplementary Figure 12. Characterization of the XPS results of AGC. a** XPS survey spectrum of AGC, and the corresponding spectrum of **b** *O 1s* and **c** *Gd 4d*. Black solid line and red dashed line corresponded to the raw and fitted curve, respectively.


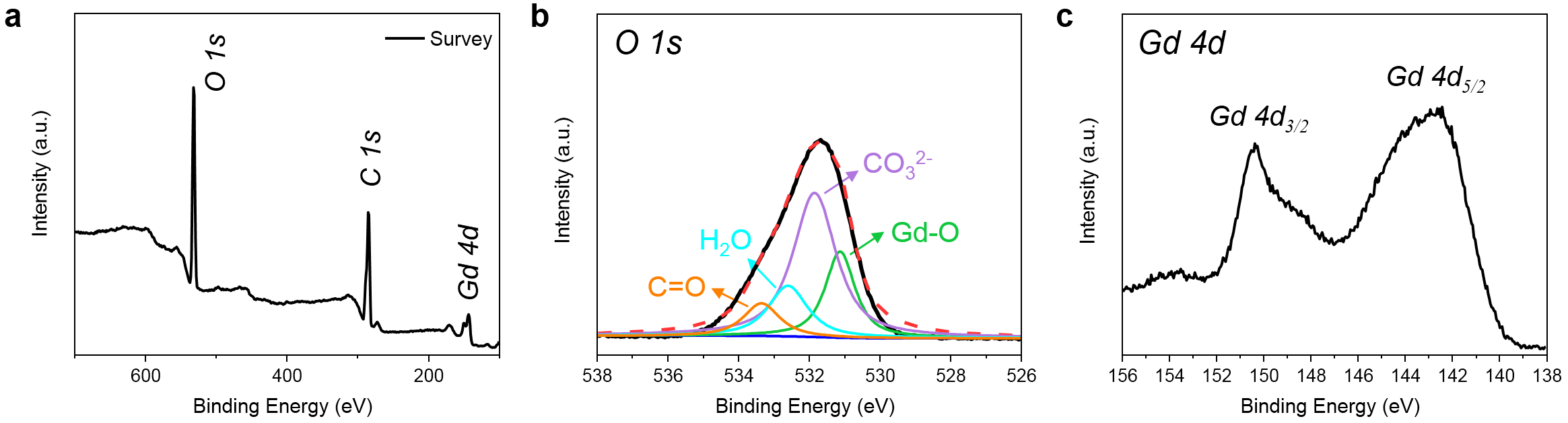


**Supplementary Figure 13. Characterization of the XPS results of AGC-PAA. a** XPS survey spectrum of AGC-PAA, and the corresponding spectrum of **b** *O 1s* and **c** *Gd 4d*. Black solid line and red dashed line corresponded to the raw and fitted curve, respectively.


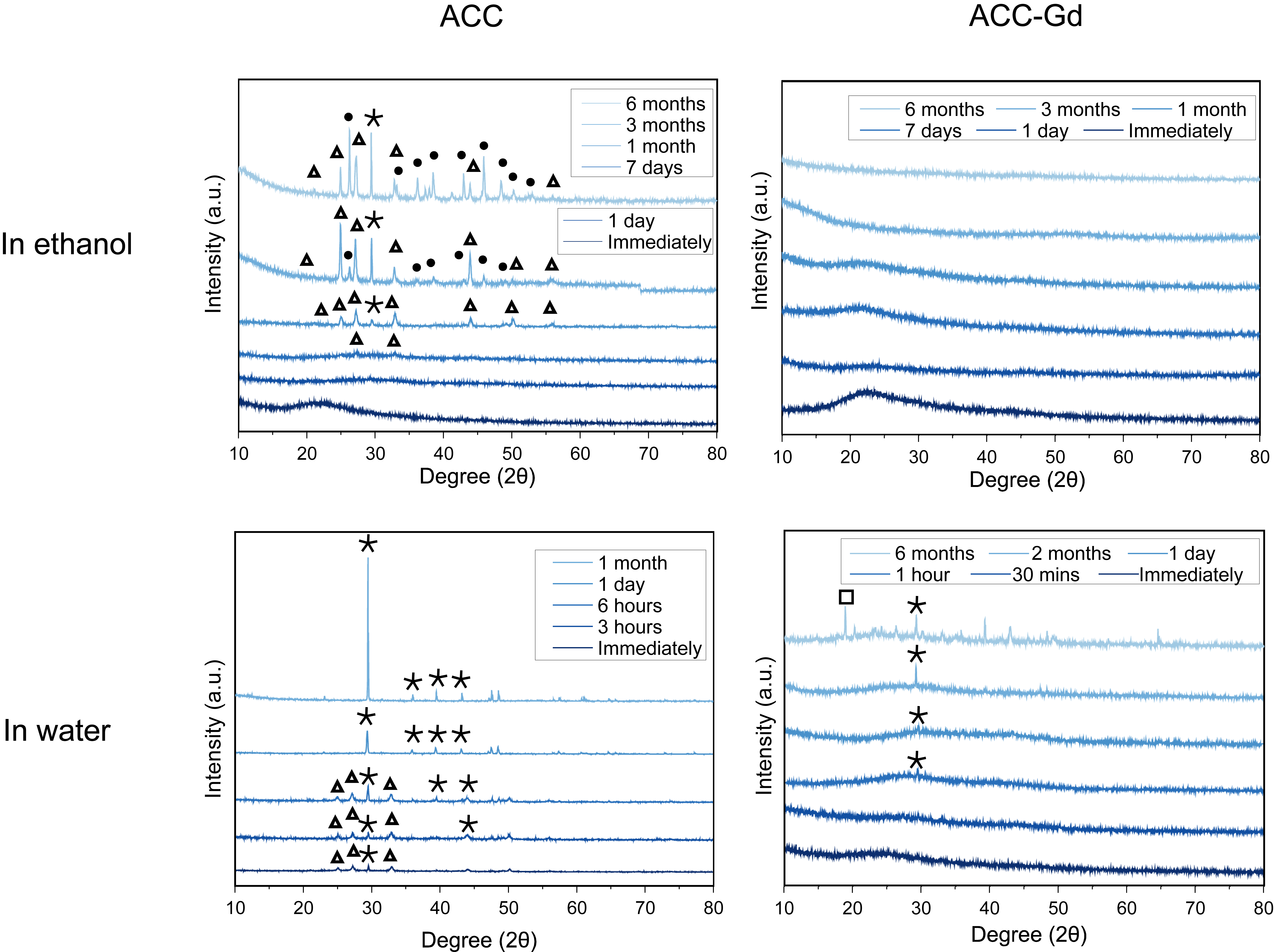


**Supplementary Figure 14.** **Characterization of the XRD results of ACC and ACC-Gd.** XRD patterns of ACC and ACC-Gd obtained from different stages in ethanol, water. The signs represent the characteristic peak of diffraction patterns from the PDF card (vaterite (Δ, PDF: 33-0268); calcite (*, PDF: 05-0586); Aragonite (●, PDF: 24-0025); gadolinium carbonate hydrate (□, PDF: 37-0559) ).


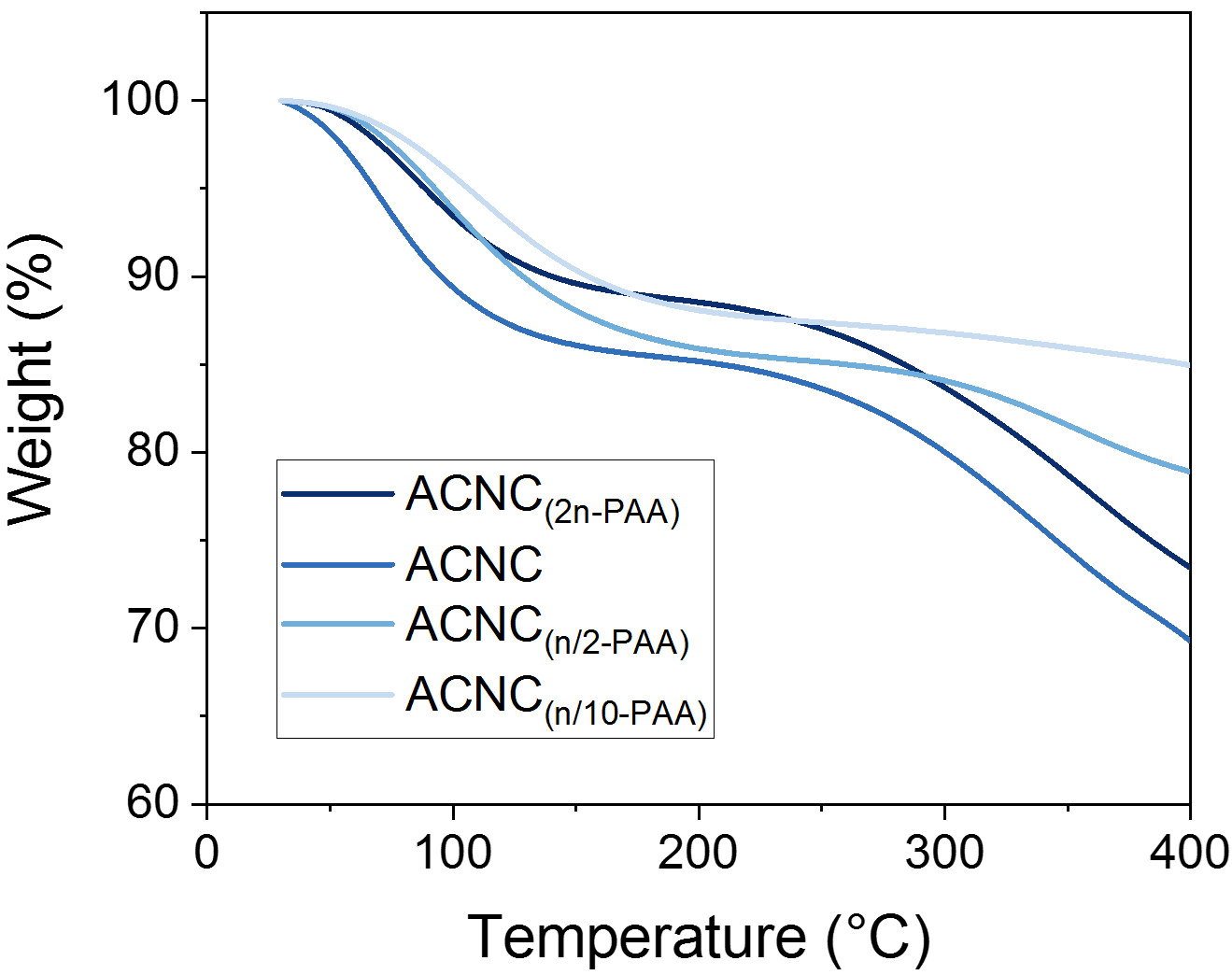


**Supplementary Figure 15.** **Characterization of the TG results of a series of control samples.** TGA of ACNC, ACNC_(2n-PAA)_, ACNC_(n/2-PAA)_ and ACNC_(n/10-PAA)_ powder under an N_2_ atmosphere with a heating rate of 10 °C min^-1^.


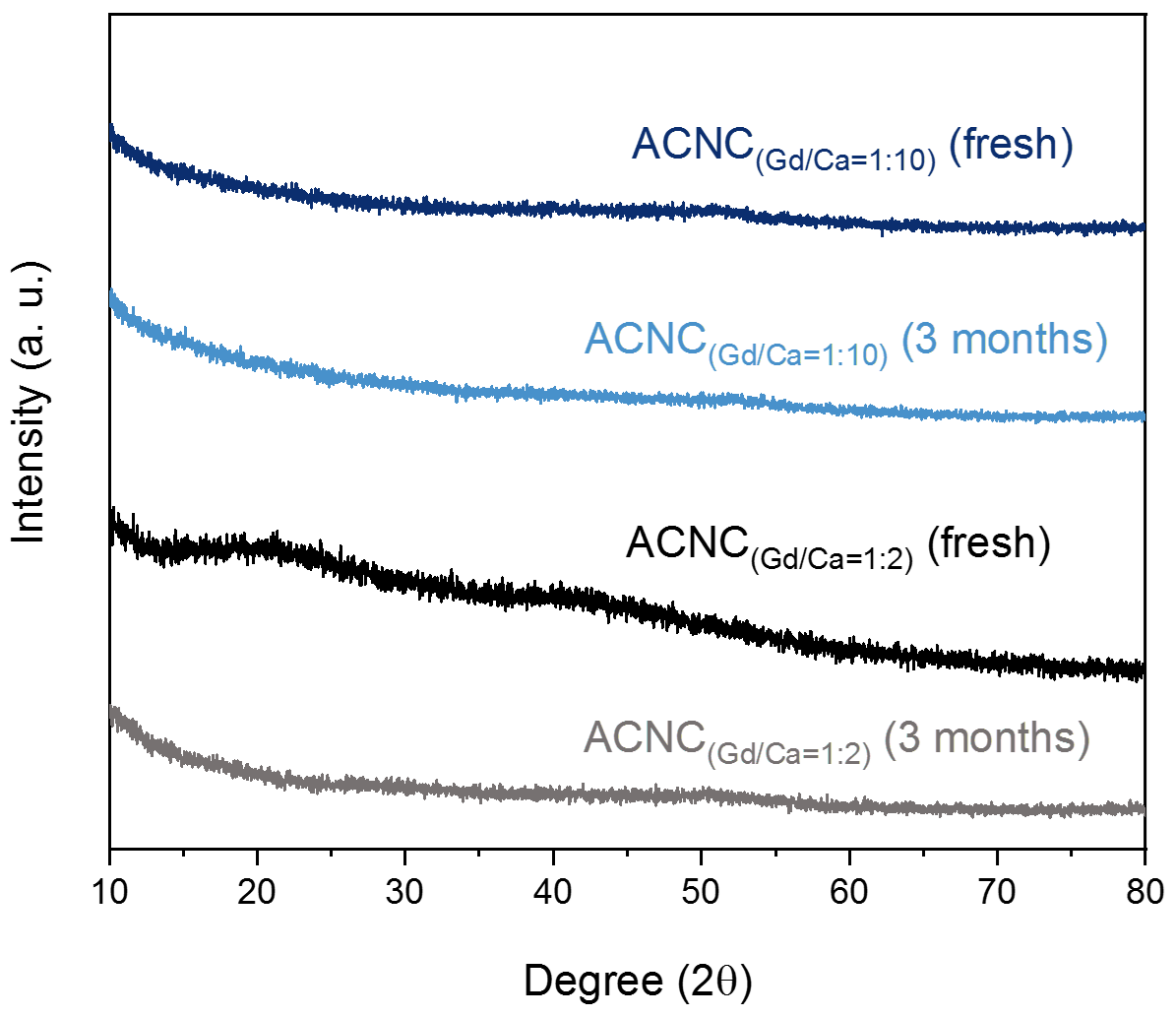


**Supplementary Figure 16. Characterization of the XRD results of a series of control samples.** XRD patterns of fresh ACNC_(Gd/Ca=1:10)_ and ACNC_(Gd/Ca=1:2)_, and them staying in aqueous solution for 3 months.


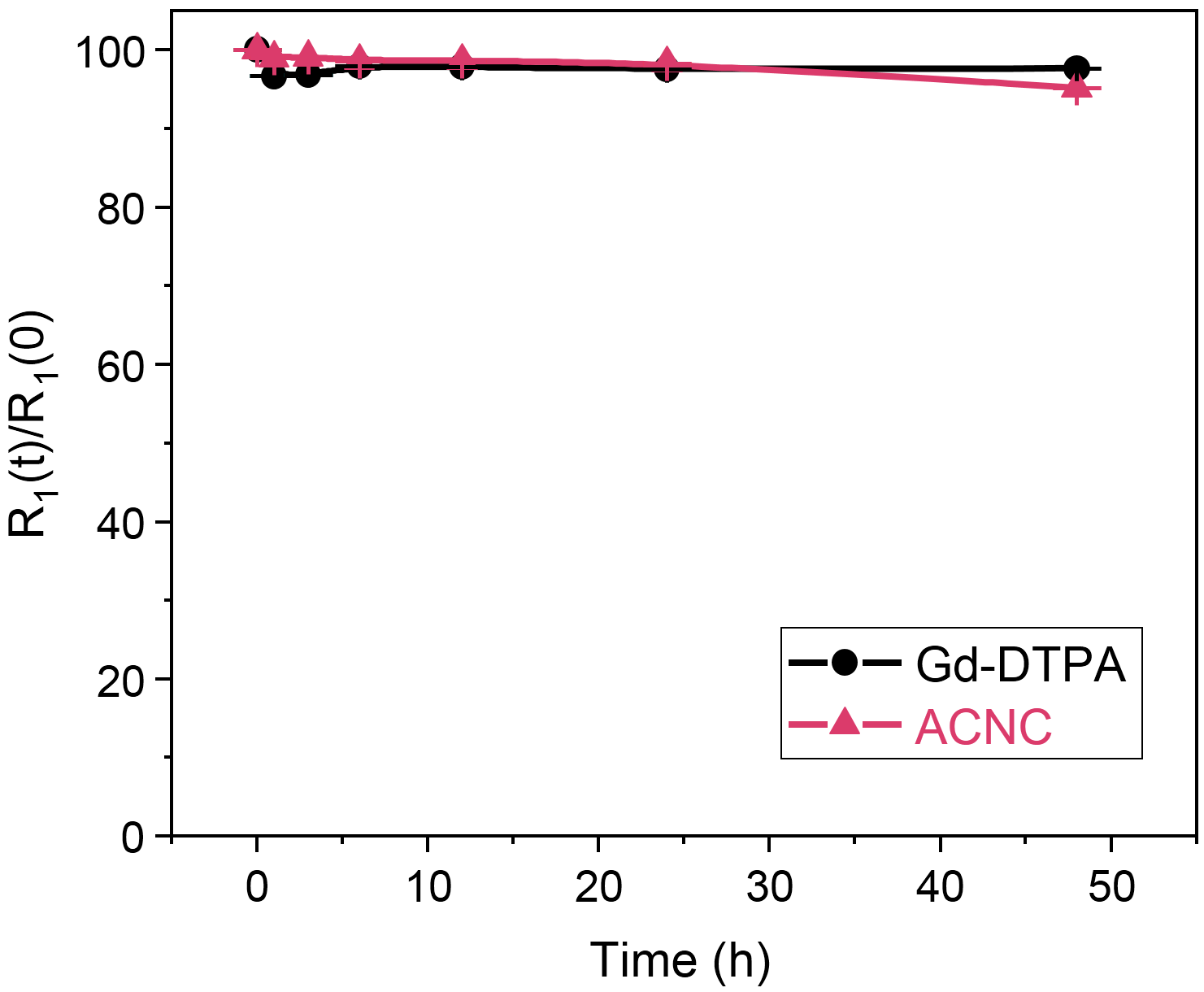


**Supplementary Figure 17.** **Ligand competition assay.** Evolution of relative R_1_ values in a competition test from the zero time to each time (R_1_(t)/R_1_(0)) for ACNC and Gd-DTPA. DTPA served as the competing ligand.


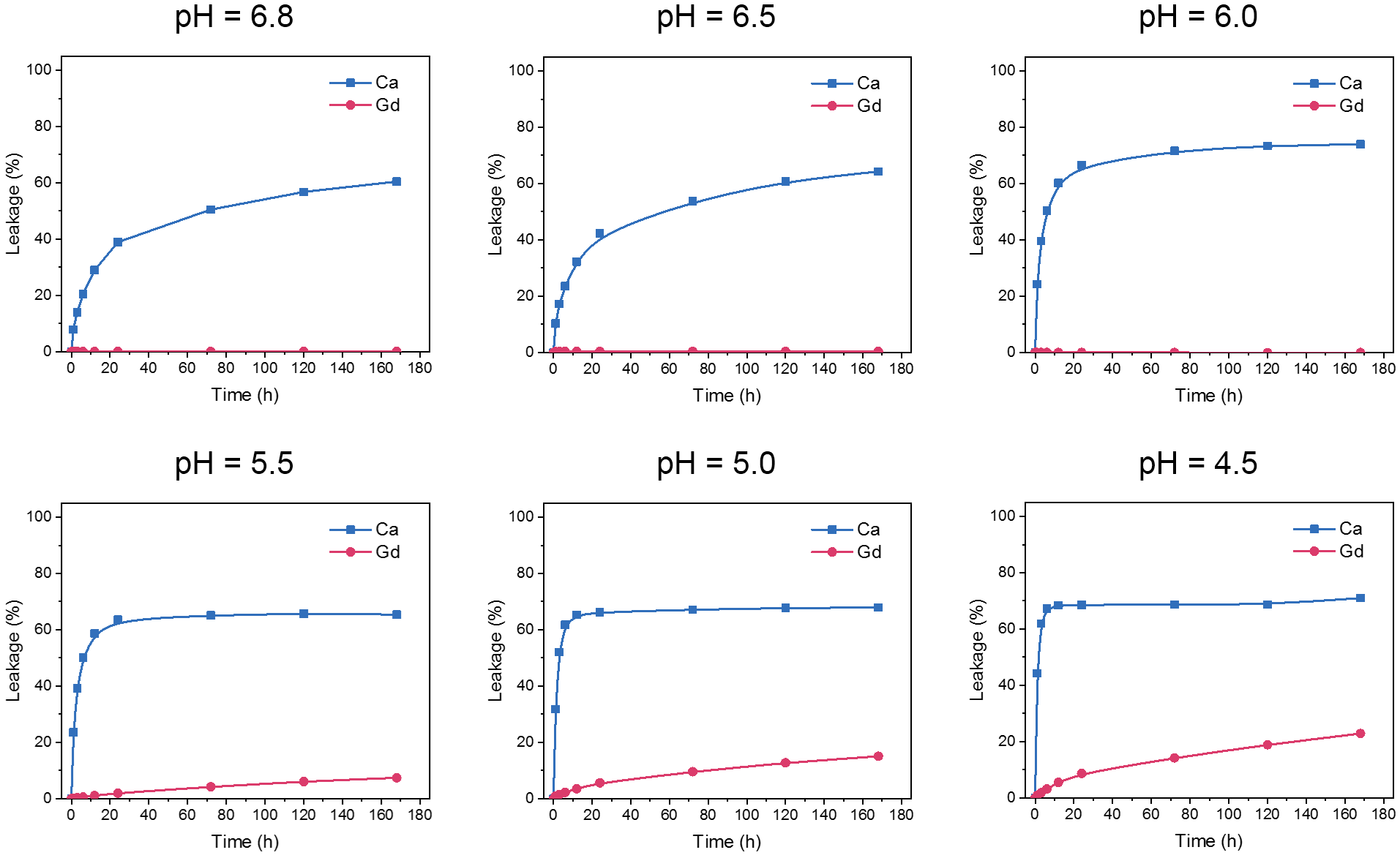


**Supplementary Figure 18.** **Degradation studies.** Cumulative leakage of free calcium and gadolinium ions from ACNC in phosphate buffers (pH 6.8, 6.5, and 6.0) and acetate buffers (pH 5.5, 5.0, and 4.5) at 37 °C within 7 days.


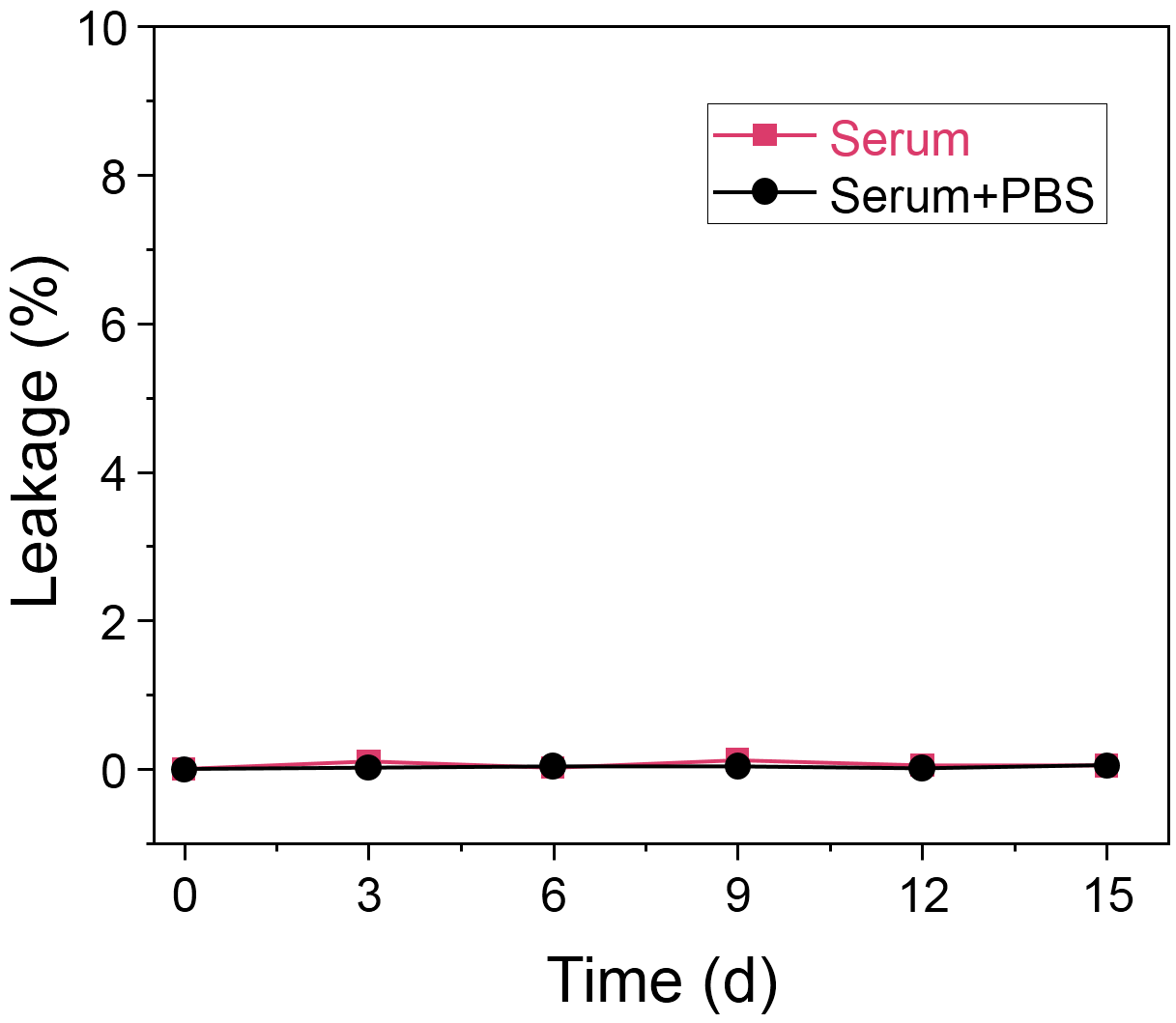


**Supplementary Figure 19.** **Leakage studies.** Analysis of the leakage of free gadolinium ion from ACNC dispersed in human serum and human serum with supplemented additional phosphate by means of ICP-MS.


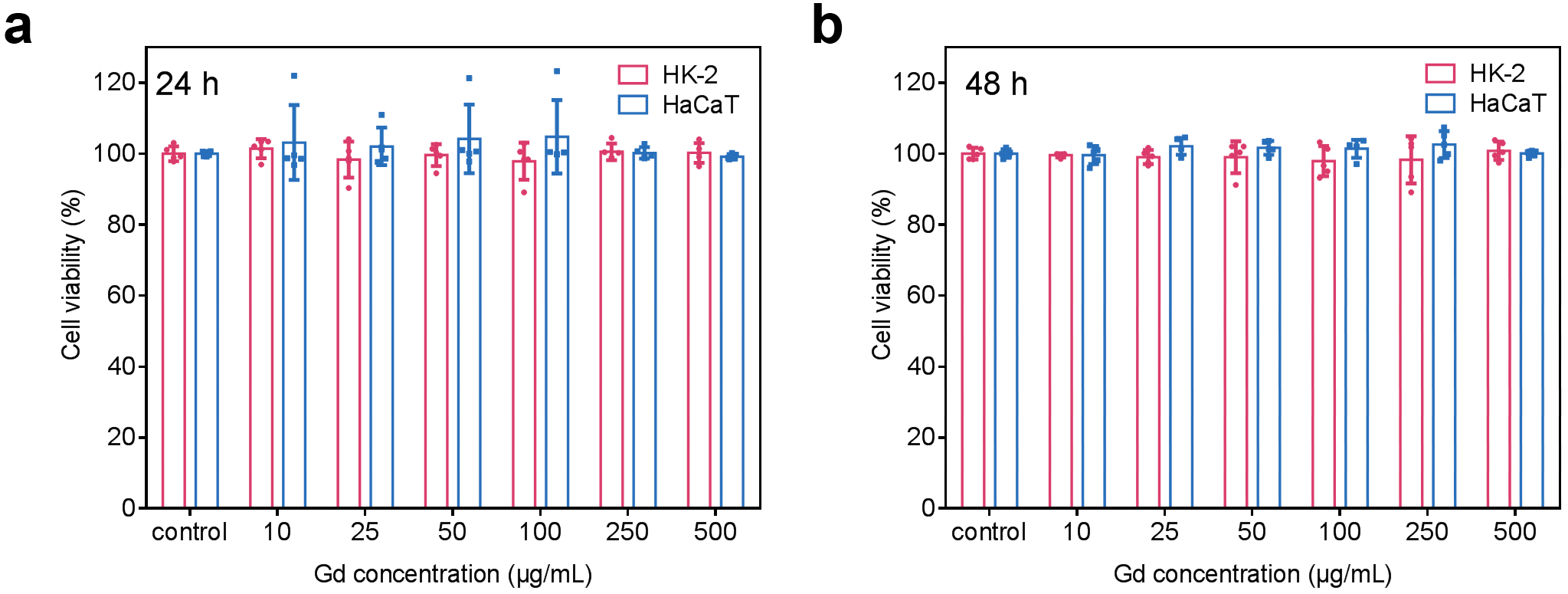


**Supplementary Figure 20. Cytotoxicity studies.** MTT results of ACNC evaluated on HK-2 and HaCaT cells at **a** 24 h and **b** 48 h (n = 3 independent experiments). The data show means ± SD.


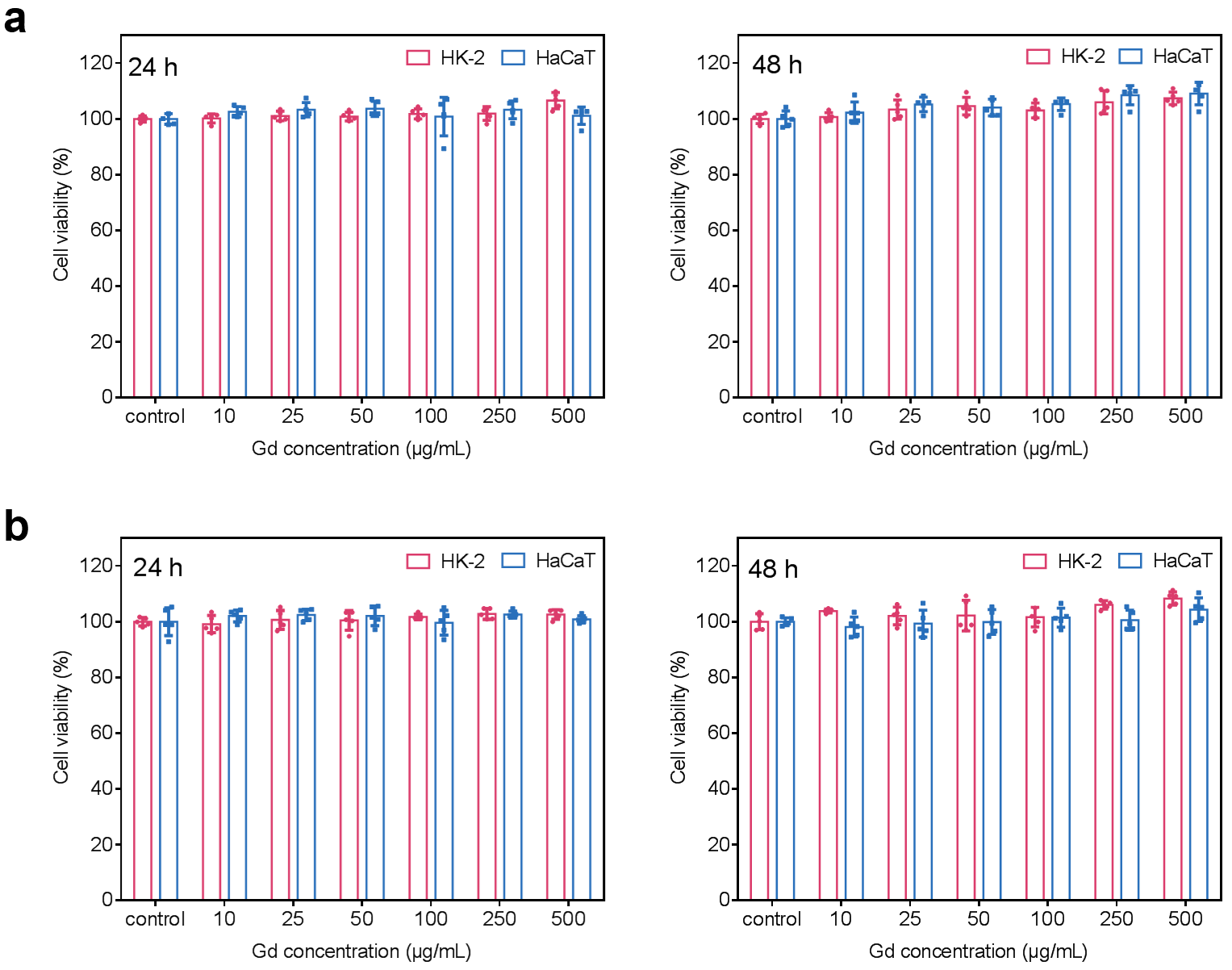


**Supplementary Figure 21. Cytotoxicity studies.** MTT results of **a** ACNC_(Gd/Ca=1:10)_ and **b** ACNC_(Gd/Ca=1:2)_ evaluated on HK-2 and HaCaT cells at 24h and 48 h (n = 3 independent experiments). The data show means ± SD.


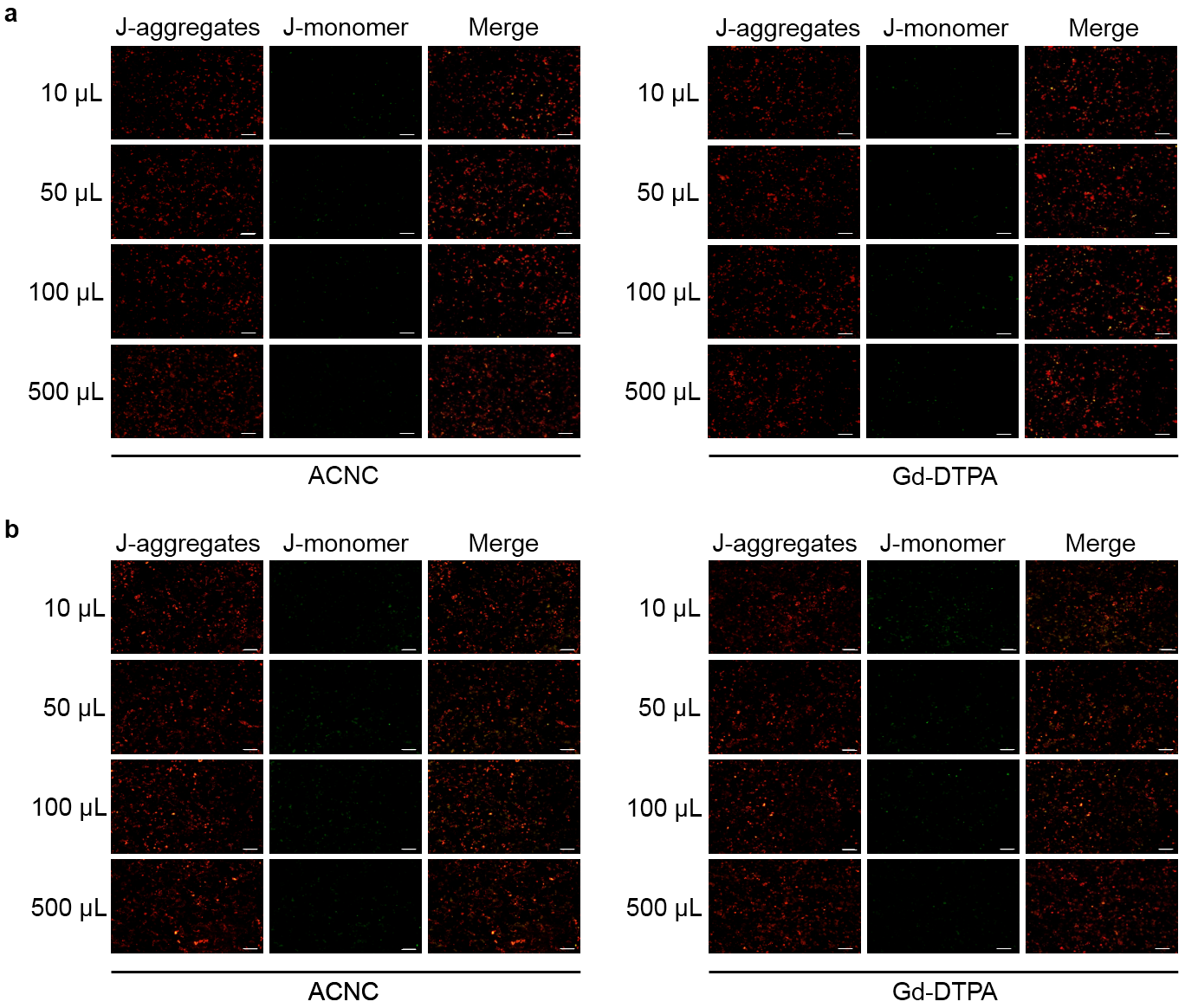


**Supplementary Figure 22. Mitochondria membrane potential study.** CLSM images of HK-2 cells stained with JC-1 after treatment with ACNC or Gd-DTPA for **a** 6 h and **b** 12 h, respectively, scale bar = 50 μm. A representative image of three individual experiments is shown.


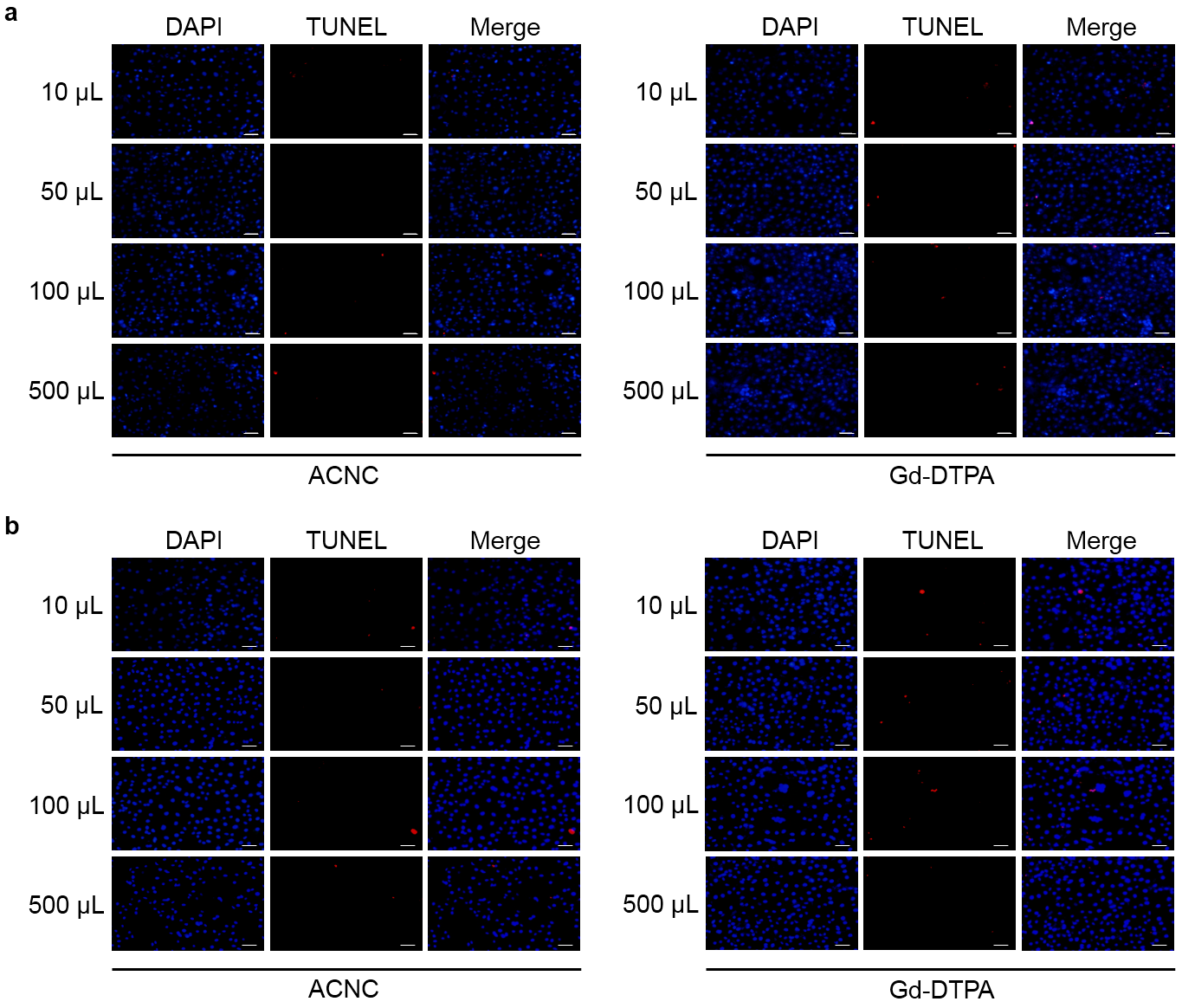


**Supplementary Figure 23. Cytocompatibility study.** Representative TUNEL immunofluorescent images of HK-2 cells separately incubated with ACNC or Gd-DTPA for **a** 24 h and **b** 48 h, scale bar = 50 μm. A representative image of three individual experiments is shown.


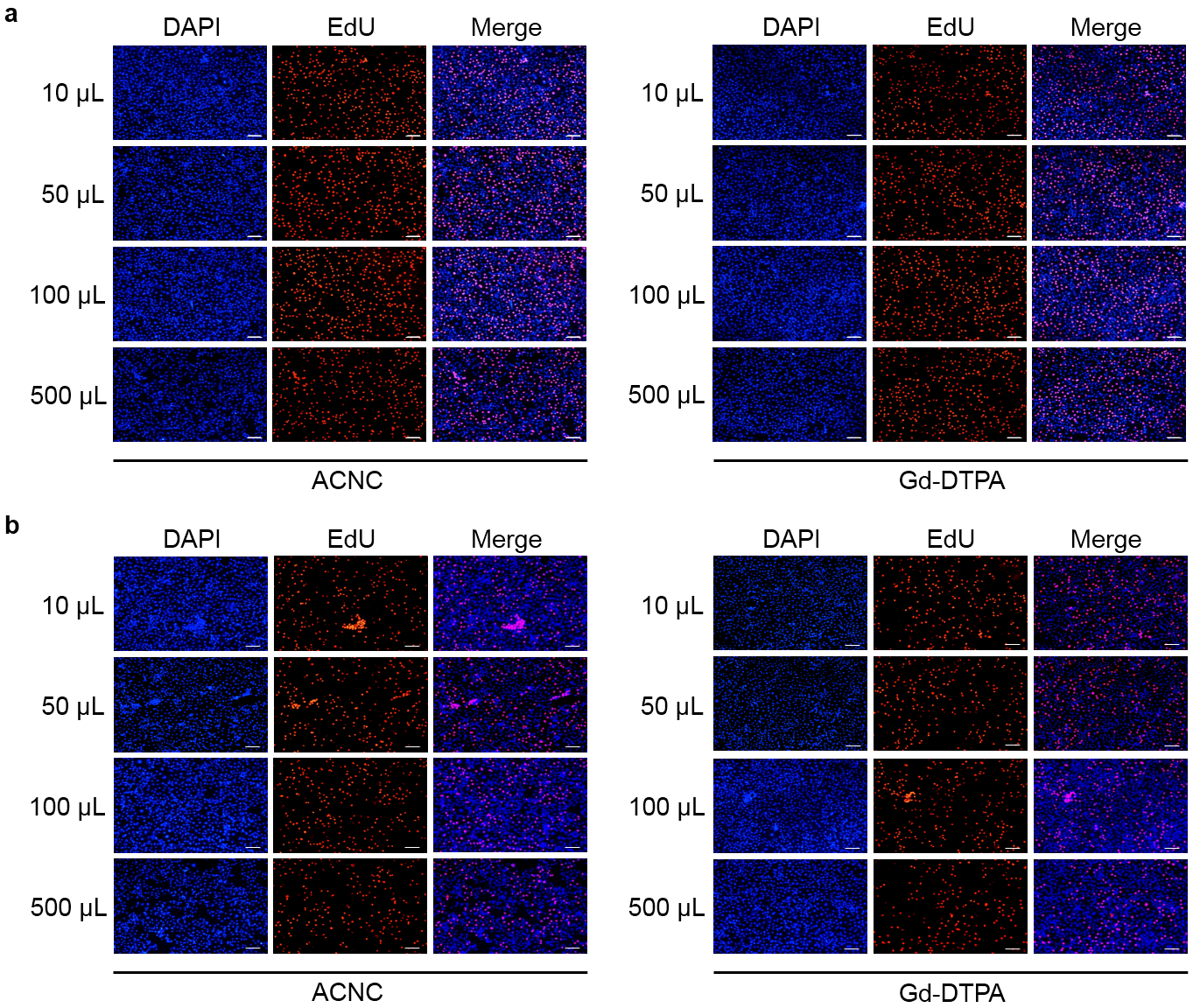


**Supplementary Figure 24. Cell proliferation study.** Cell proliferation in HK-2 cells by EdU cell proliferation assay, scale bar = 50 μm. The cells were incubated with ACNC or Gd-DTPA for **a** 24 h and **b** 48 h, respectively. A representative image of three individual experiments is shown.


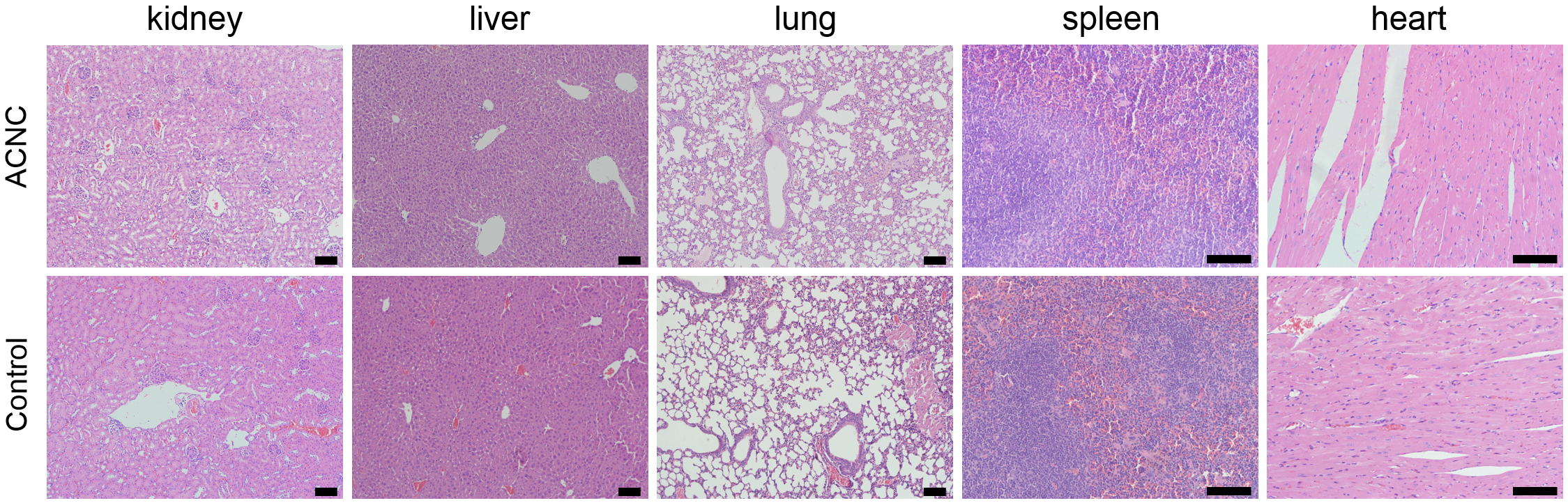


**Supplementary Figure 25.** **Histological analyses.** Histological analyses of tissues of mice including kidney, liver, lung, spleen and heart 2 weeks after the intravenous injection of ACNC. The scale bar is 100 μm. A representative image of three individual experiments is shown.


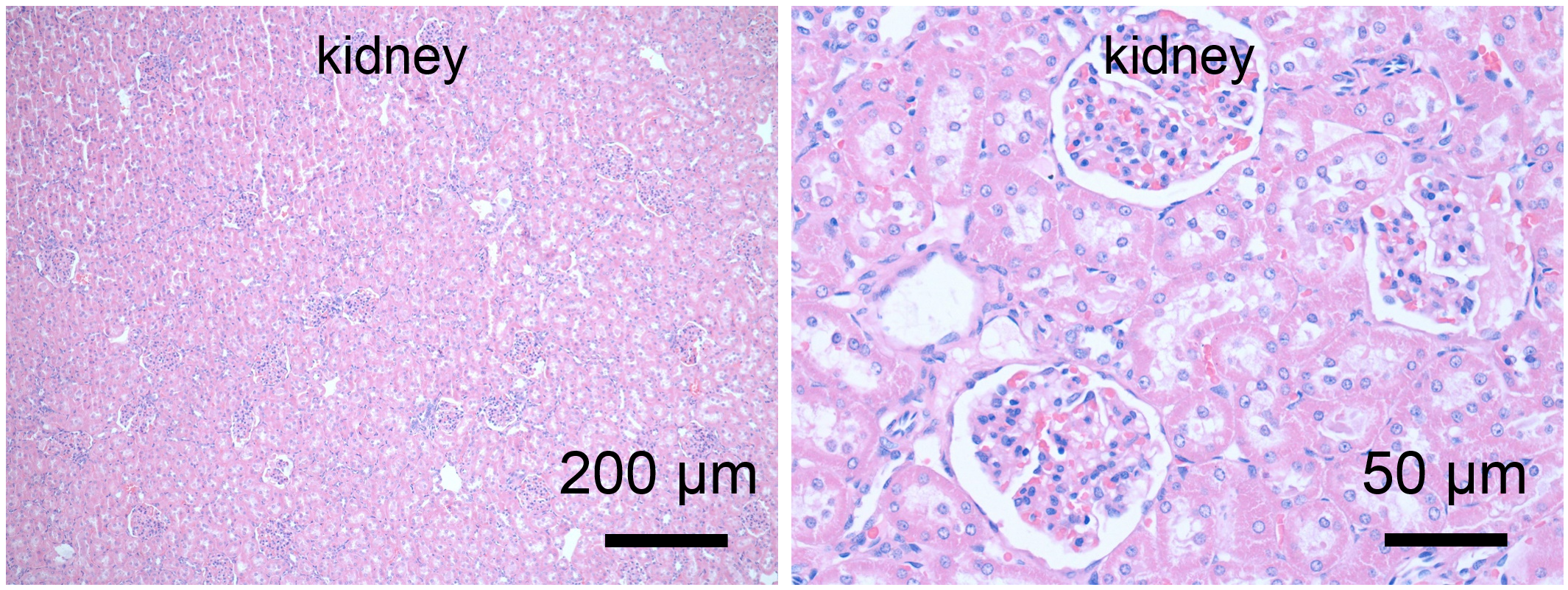


**Supplementary Figure 26.** **Histological analyses.** Histological analyses of kidney tissue of rabbit, 30 minutes after the intravenous injection of ACNC. A representative image of three individual experiments is shown.


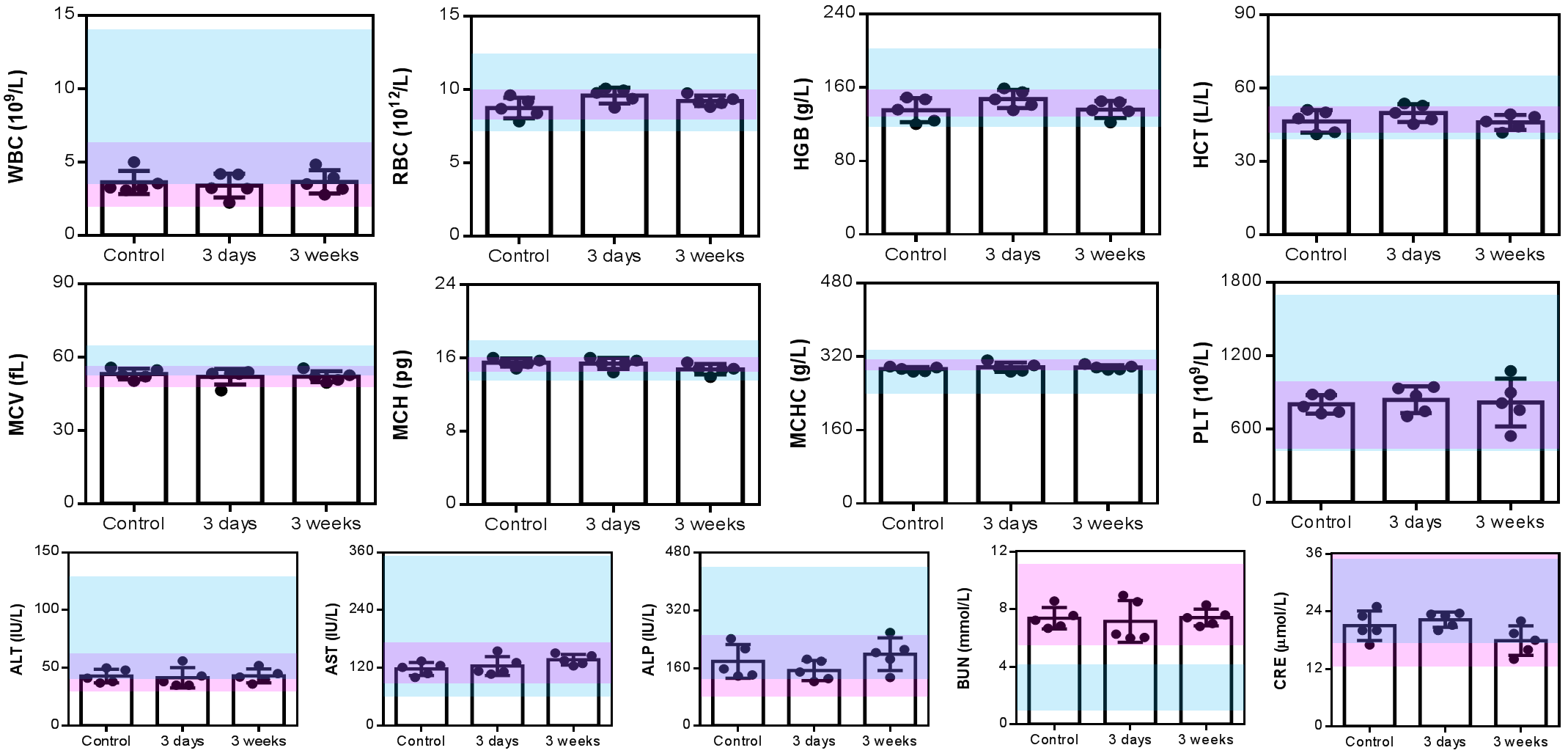


**Supplementary Figure 27. Blood biochemical examination and blood routine examination in mouse.** Major hematologic and biochemical indices of liver and kidney were examined 3 days and 3 weeks after the intravenous injection of ACNC in BALB/c mice (n = 5 biologically independent animals), and the intravenous injection of normal saline was set as control. The data show means ± SD. Major indices were listed, including white blood cells (WBC), red blood cells (RBC), hemoglobin (HGB), hematocrit (HCT), mean corpuscular volume (MCV), mean corpuscular hemoglobin (MCH), mean corpuscular hemoglobin concentration (MCHC), and platelets (PLT), alanine transaminase (ALT), aspartate transaminase (AST), alkaline phosphatase (ALP), blood urea nitrogen (BUN), and creatinine (CRE). Reference ranges of hematologic and biochemical indices of healthy Balb/c mice (the blue labelled region) were obtained from Charles River Laboratories (http://www.criver.com/). Meanwhile, the reference normal range from 30 healthy Balb/c mice in our group was marked by pink.


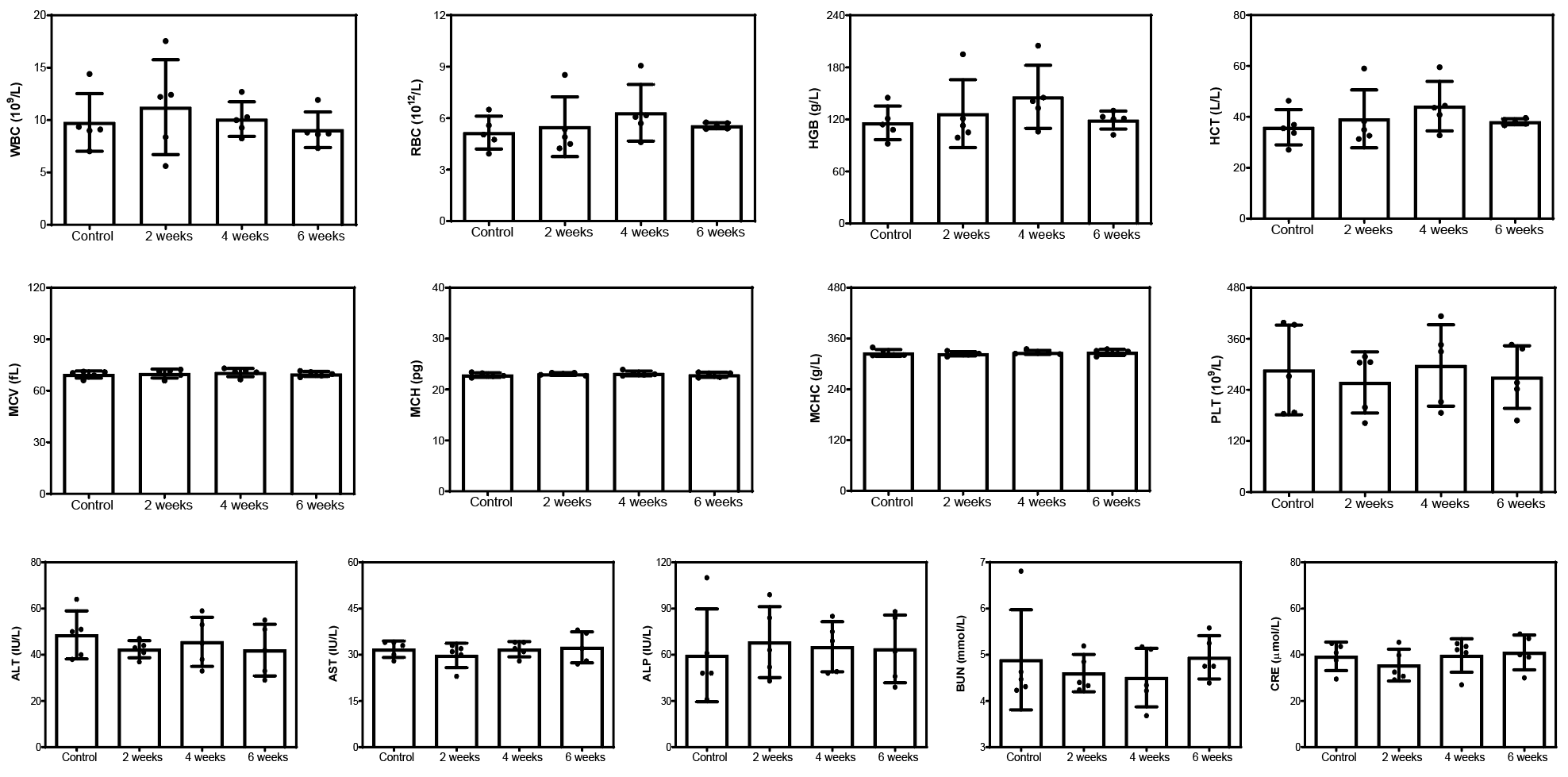


**Supplementary Figure 28. Blood biochemical examination and blood routine examination in beagle dog at a high dose (9 mg/kg bw).** Major hematologic and biochemical indices of liver and kidney were examined every 2 weeks after the intravenous injection of ACNC in beagle dogs (n = 5 biologically independent animals), and the intravenous injection of normal saline was set as control. The data show means ± SD. Major indices were listed, including white blood cells (WBC), red blood cells (RBC), hemoglobin (HGB), hematocrit (HCT), mean corpuscular volume (MCV), mean corpuscular hemoglobin (MCH), mean corpuscular hemoglobin concentration (MCHC), and platelets (PLT), alanine transaminase (ALT), aspartate transaminase (AST), alkaline phosphatase (ALP), blood urea nitrogen (BUN), and creatinine (CRE).


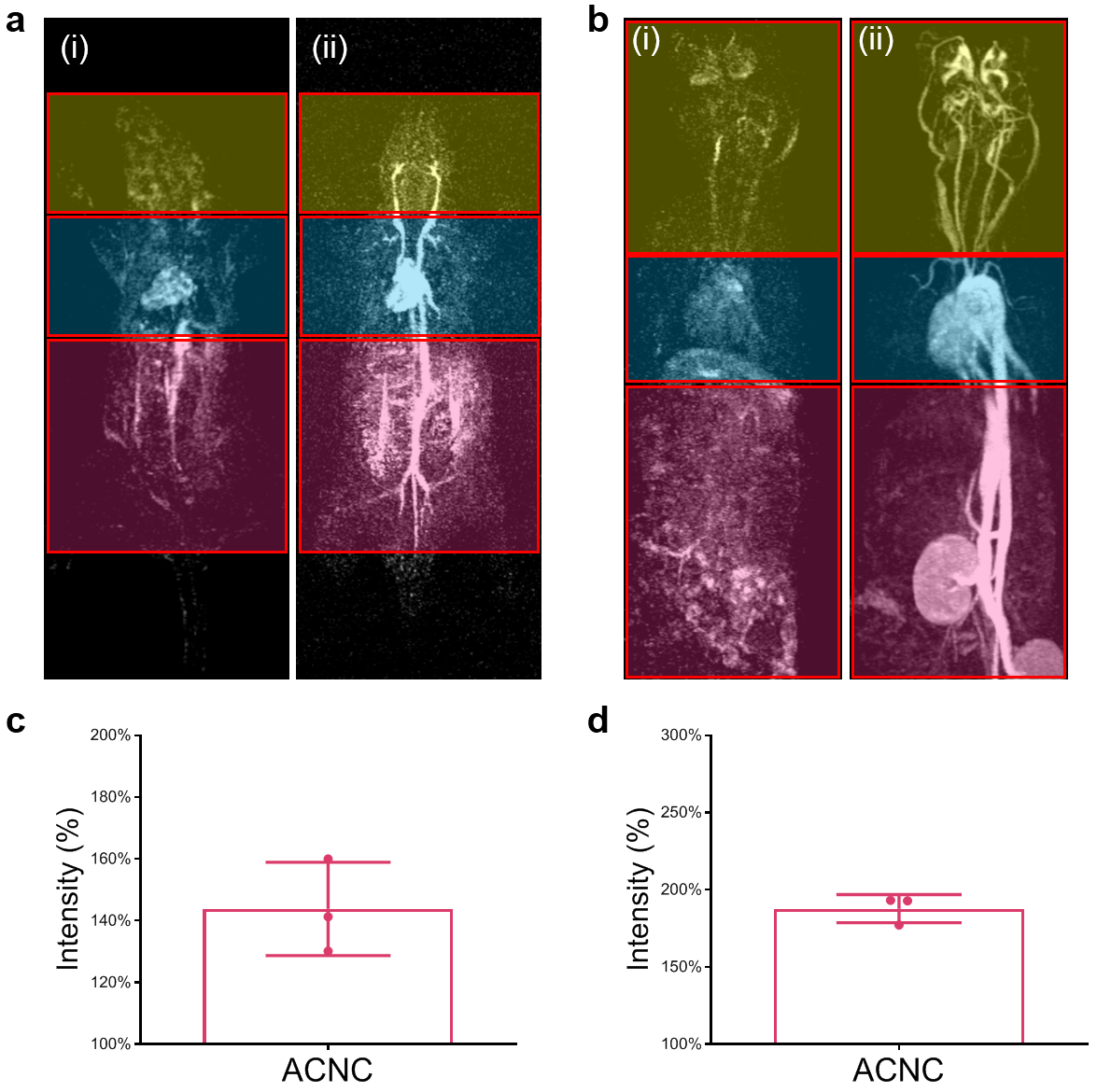


**Supplementary Figure 29. Semiquantitative analysis.** Contrast enhanced MR angiography (MRA) images on **a** rat and **b** rabbit, and three statistical areas marked by yellow, blue, red box, respectively. Semiquantitative analysis of the enhancement of signal intensity of (ii) ACNC groups in comparison to those in (i) Gd-DTPA groups on **c** rat and **d** rabbit at the immediate (IM) time point from three areas (n = 3 independent areas). The data show means ± SD.


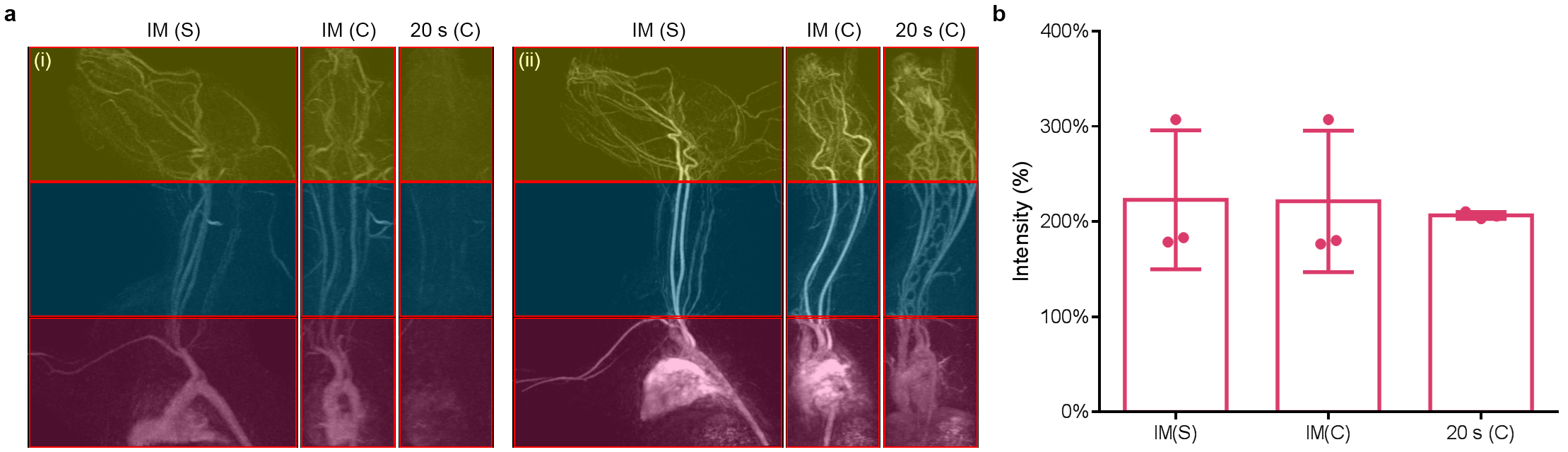


**Supplementary Figure 30. Semiquantitative analysis. a** Sagittal (S) and Coronal (C) MRA images on beagle dog and three statistical areas marked by yellow, blue, red box, respectively. **b** Semiquantitative analysis of the enhancement of signal intensity of (ii) ACNC groups in comparison to those in (i) Gd-DTPA groups on beagle dog at the immediate (IM) and 20 s time points from three areas (n = 3 independent areas). The data show means ± SD.


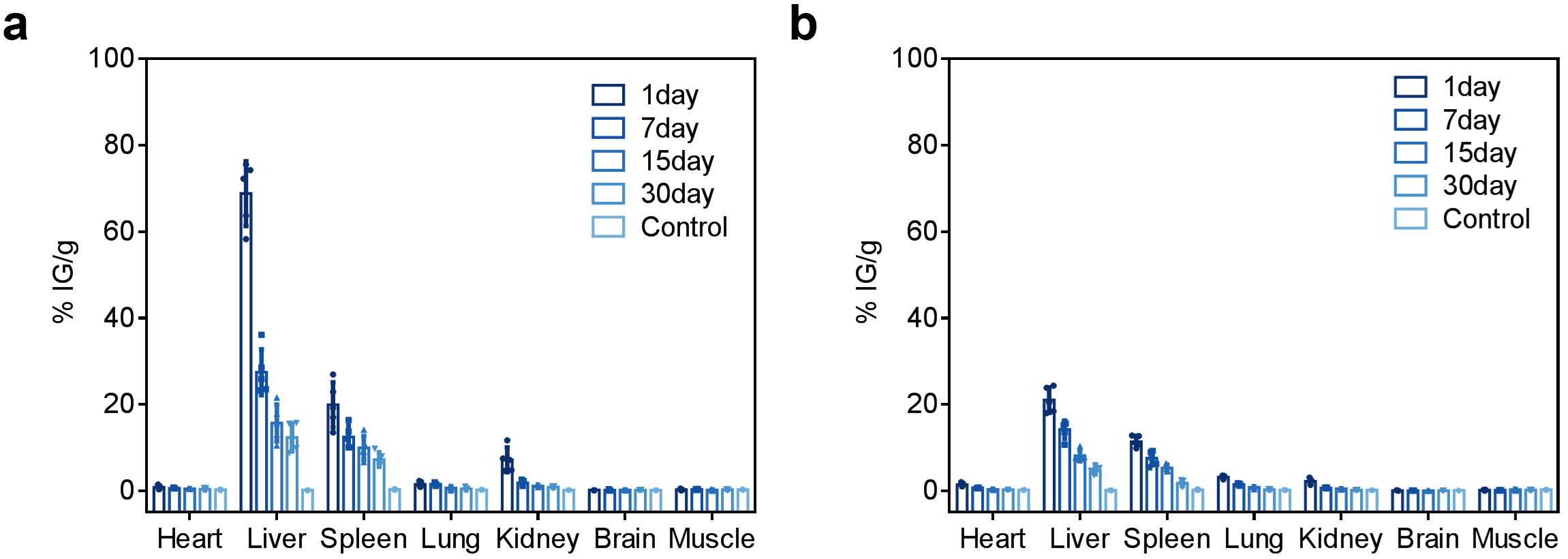


**Supplementary Figure 31. Biodistribution study.** The time dependent distribution of ACNC in organs of mice after **a** rapid manual injection or **b** normal manual injection of ACNC (n = 5 biologically independent animals). The data show means ± SD. A rapid manual injection was administered with simulated bolus injections (~1 mL/s) to mimic the rapid clinical medication of MR contrast agent via high-pressure syringe that was administrated in our *in vivo* studies.


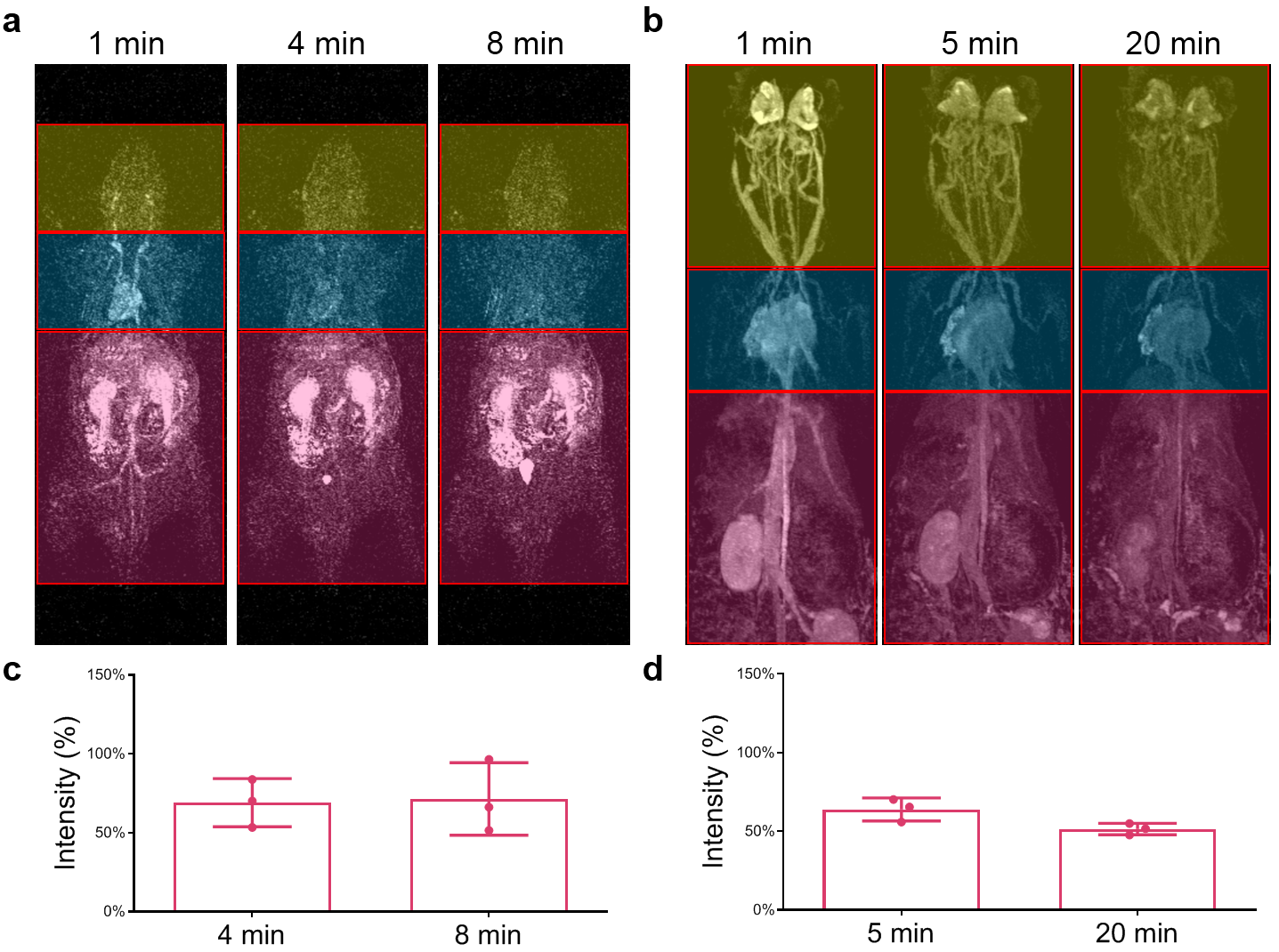


**Supplementary Figure 32. Semiquantitative analysis.** MR angiography (MRA) images on **a** rat and **b** rabbit and three statistical areas marked by yellow, blue, red box, respectively. Semiquantitative analysis of the residual signal intensity of ACNC groups on **c** rat and **d** rabbit at different time points in comparison to those at 1 min from three areas (n = 3 independent areas). The data show means ± SD.


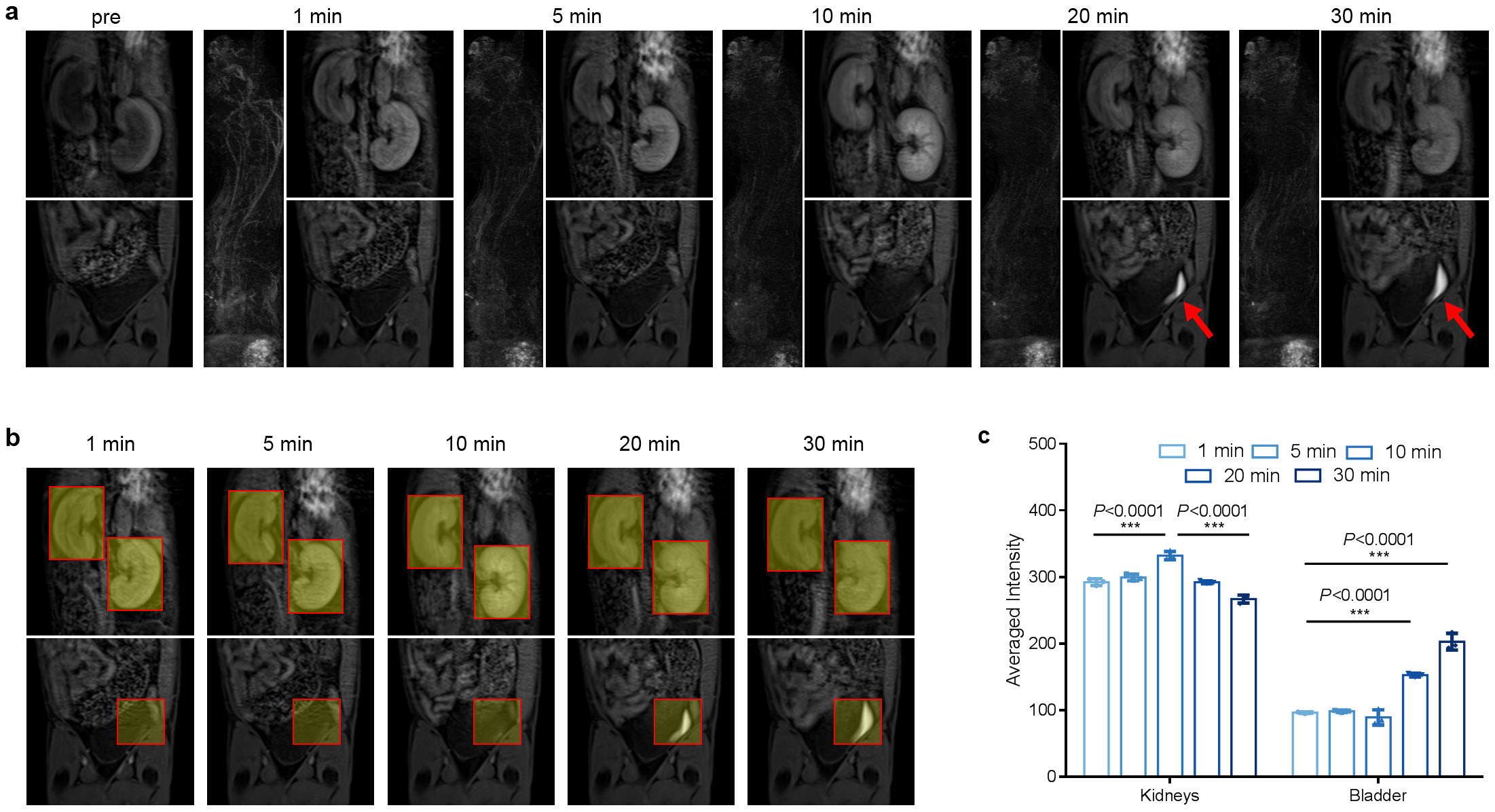


**Supplementary Figure 33. Semiquantitative analysis.** MR angiography and rapid renal clearance of ACNC. **a** MR angiography on beagle dog at different time points after the intravenous injection of ACNC to analyse its dynamic distribution. The kidney continues to brighten within 10 minutes and the bladder (the red arrow) brightened obviously after 10 minutes. **b** selected areas within the yellow box and **c** semiquantitative analysis of selected areas to analyse ACNC’s dynamic distribution in kidneys and bladder region, respectively (n = 3 independent calculations). The data show means ± SD. One-way ANOVA with Tukey’s multiple comparisons test was used for **c** to calculate the *P* value (****P*<0.001).


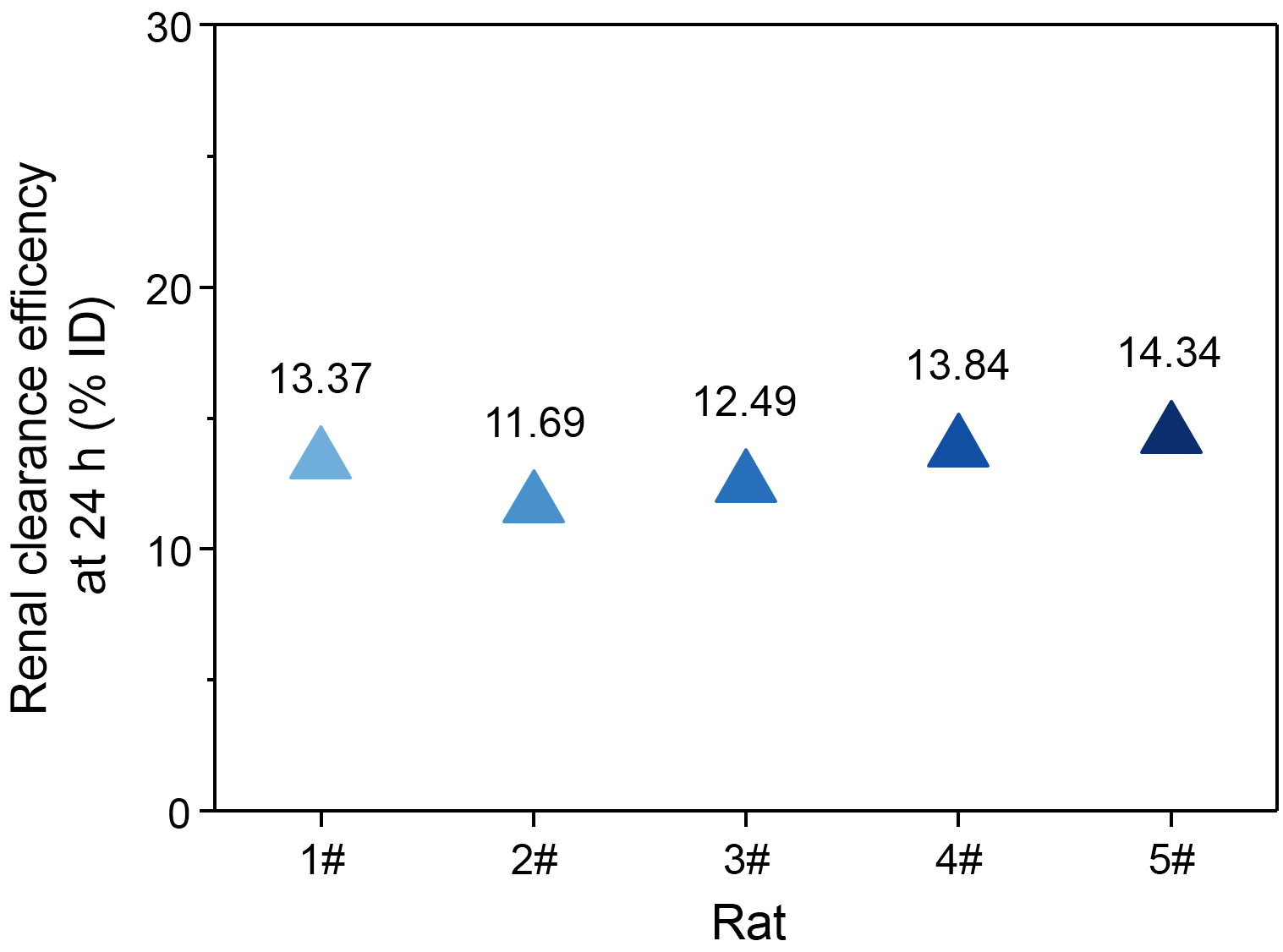


**Supplementary Figure 34. Renal clearance efficiencies.** Renal clearance efficiencies of ACNC in five rats at 24h.

**Supplementary Table 1** EXAFS fit values for ACC standard, ACC-PAA, ACC-Gd and ACNC samples.

| **Sample** | **N**  **(Coordination number)** | **R (Å)** | **Debye-Waller (10^2^ Å^2^)** |
| --- | --- | --- | --- |
| ACC | 5.2 ± 0.8 | 2.41 ± 0.03 | 0.8 ± 0.1 |
| ACC-PAA | 5.9 ± 0.9 | 2.40 ± 0.03 | 0.7 ± 0.1 |
| ACC-Gd | 5.7 ± 0.9 | 2.42 ± 0.03 | 1.0 ± 0.1 |
| ACNC | 6.0 ± 0.9 | 2.41 ± 0.03 | 0.9 ± 0.1 |

**Supplementary Table 2** Linear combination results between standards ACC-PAA and PAA-Ca and samples PAA-Ca/Gd, ACC-Gd and ACNC.

| Standard | ACC-PAA | PAA-Ca |
| --- | --- | --- |
| Samples | R^2^ | R^2^ |
| PAA-Ca/Gd | 0.00% | 100.00% |
| ACC-Gd | 93.80% | 6.20% |
| ACNC | 95.70% | 4.30% |

**Supplementary Table 3** Details of final product ACNC and a series of control samples. ‘N/A’ represents the absence of a component, while ‘+’ represents the presence.


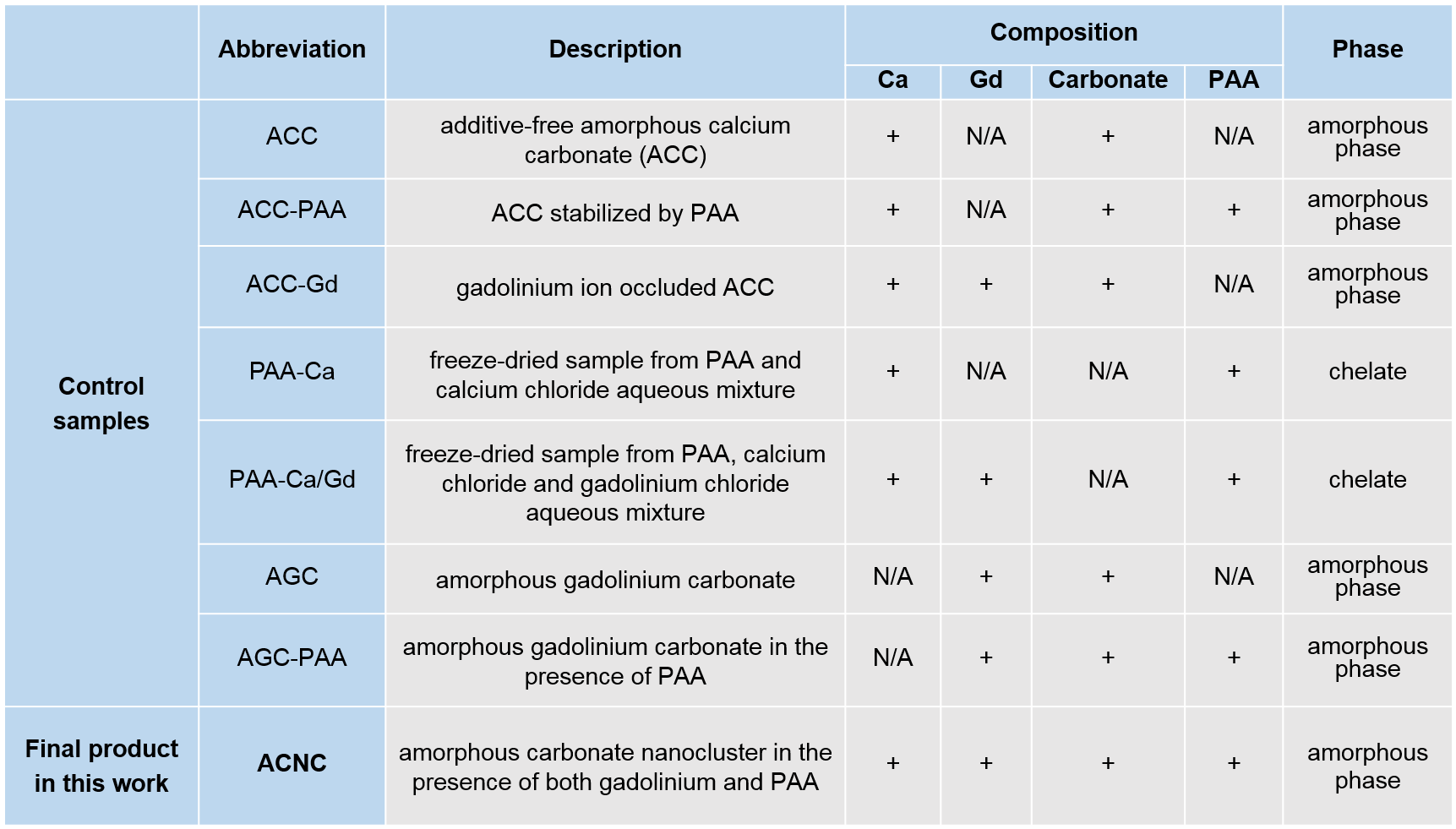


**Supplementary Table 4** Results of AUC experiments. Results of AUC experiments using 2-dimensional spectrum analysis (2-DSA) for the determination of the cluster hydration calculated for spherical clusters via the Perrin coefficient[^1^](#_ENREF_1). Absorption optics was used for ACNC detection via PAA (230 nm). s = sedimentation coefficient, D = diffusion coefficient, ρ= density of the cluster (n=3) and δ = hydration.

| Sample | s  (10^-13^s) | D  10^-10^ (m^2^/s) | d_H_ (nm) | M  (g/mol) | ρ (g/cm^3^) | $\bar{v}$ (cm^3^/g) | f/f_0_ | δ (g_H2O_/g_Solute_) |
| --- | --- | --- | --- | --- | --- | --- | --- | --- |
| ACNC1 | 1.19 | 2.86 | 1.5 | 2030 | 2.0171 | 0.4958 | 1.90 | 2.90 |
| ACNC2 | 1.20 | 2.80 | 1.5 | 2200 | 2.0045 | 0.4989 | 2.00 | 3.49 |
| ACNC3 | 1.17 | 2.60 | 1.6 | 2460 | 1.8157 | 0.5508 | 1.90 | 3.22 |
| Average | 1.19 | 2.75 | 1.5 | 2230 | 1.9458 | 0.5152 | 1.93 | 3.20 |

**Supplementary Table 5** Water content of ACNC in solution measured by AUC (n=3).

| **Sample** | **Molar ratio of water molecules per mole of CaCO_3_** | **Molar ratio of water molecules per mole of Gd** |
| --- | --- | --- |
| ACNC1 | 7.6 | 19.8 |
| ACNC2 | 10 | 26.0 |
| ACNC3 | 8.9 | 23.2 |
| **Average** | **8.8** | **23.0** |

**Supplementary Table 6** The time dependent distribution of ACNC in plasma of mouse and beagle dog within 24 h (n = 5 biologically independent animals).

**
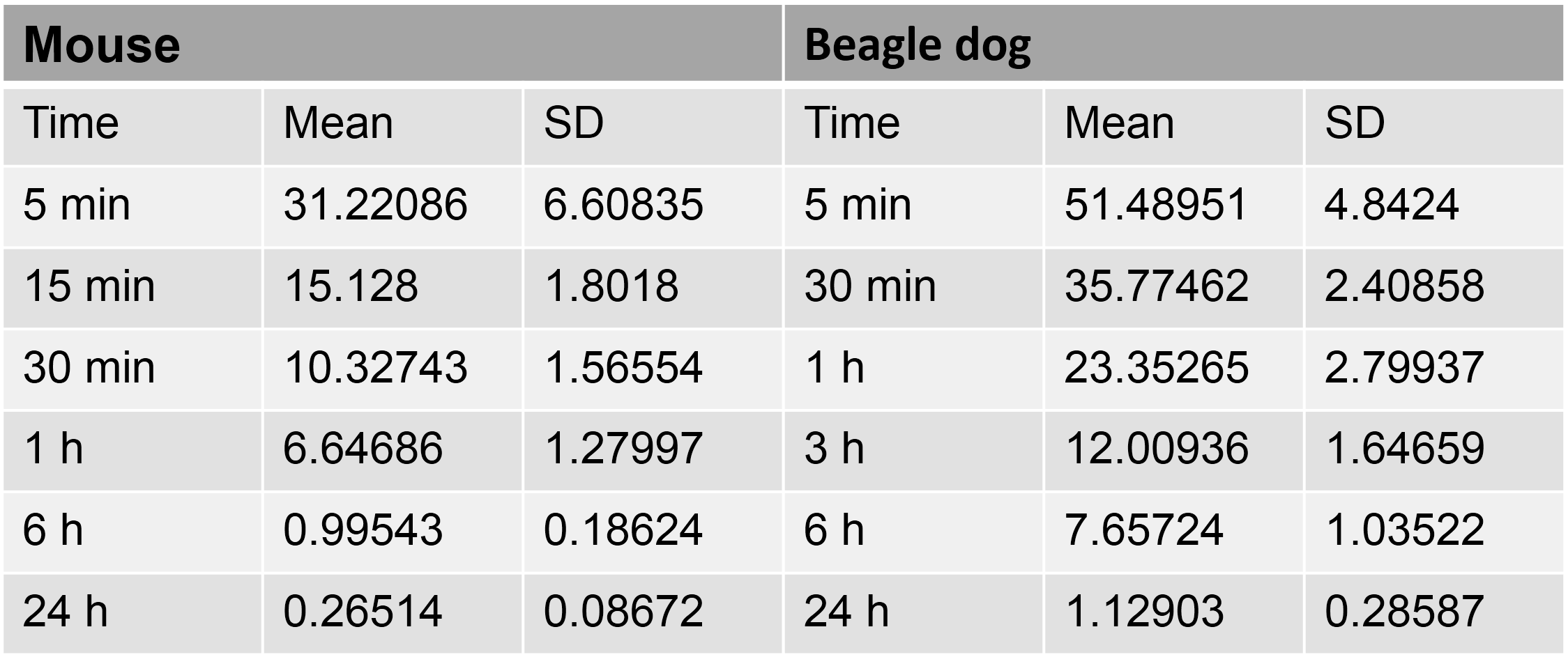
**

**Supplementary References**

1. Schuck, P., Rossmanith, P. Determination of the sedimentation coefficient distribution by least-squares boundary modeling. *Biopolymers* **54,** 328-341 (2000).
